# Ultrafast Carrier and Lattice Cooling in Ti_2_CT_*x*_ MXene Thin Films

**DOI:** 10.1021/acs.nanolett.4c04583

**Published:** 2024-11-22

**Authors:** Tong Wang, Chengning Yao, Ruoyu Gao, Martin Holicky, Beier Hu, Sihui Liu, Shuwei Wu, Hyunho Kim, Haoqing Ning, Felice Torrisi, Artem A. Bakulin

**Affiliations:** † Department of Chemistry and Centre for Processible Electronics, Imperial College London, London W12 0BZ, United Kingdom; ‡ Dipartimento di Fisica e Astronomia, Universita’ di Catania and CNR-IMM (Catania Universita’), Via S. Sofia 64, 95123 Catania, Italy

**Keywords:** MXenes, ultrafast spectroscopy, carrier cooling, lattice cooling, heat dissipation

## Abstract

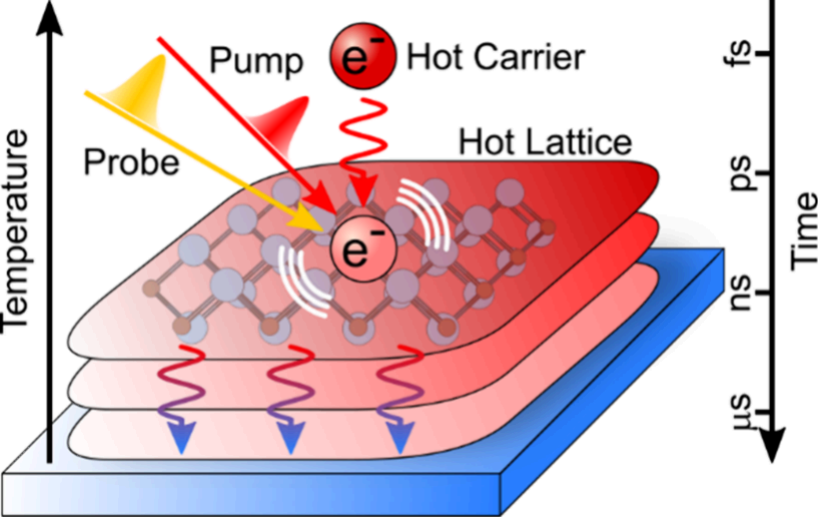

Metallic MXenes are promising two-dimensional materials for energy storage, (opto)electronics, and photonics due to their high electrical conductivity and strong light–matter interaction. Energy dissipation in MXenes is fundamental for photovoltaic and photothermal applications. Here we apply ultrafast laser spectroscopy across a broad time range (femto- to microseconds) to study the cooling dynamics of electrons and lattice in emerging Ti_2_CT_*x*_ thin films compared to widely studied Ti_3_C_2_T_*x*_ thin films. The carrier cooling time in Ti_2_CT_*x*_ is persistently ∼2.6 ps without a hot-phonon bottleneck. After hot carrier cooling is completed, the transient absorption spectra of Ti_2_CT_*x*_ MXene can be described well by the thermochromic effect. Heat dissipation in MXene thin films occurs over hundreds of nanoseconds with thermal diffusivities ∼0.06 mm^2^ s^–1^ for Ti_2_CT_*x*_ and ∼0.02 mm^2^ s^–1^ for Ti_3_C_2_T_*x*_, likely due to inefficient interflake heat transfer. Our results unravel the energy dissipation dynamics in Ti_2_CT_*x*_ films, showcasing potential applications in energy conversion.

MXenes make up an emerging family of the two-dimensional (2D) materials with a formula of M_*n+*1_X_*n*_T_*x*_, where M is an early transition metal, X is carbon and/or nitrogen, *n* = 1–4, and T represents surface functional groups (such as −OH, =O, −Cl, and −F).^1,2^ Currently, a diverse range of MXenes have been successfully synthesized and used in different applications.^3^ Ti_3_C_2_T_*x*_ MXene is one of the most studied MXenes, including theoretical explanation and applications in energy storage and (opto)electronics.^1,2,4−6^ However, other Ti-based MXenes, like Ti_2_CT_*x*_, are expected to offer better balance between mechanical and electronic properties.^7−10^

Metallic Ti_2_CT_*x*_ MXene has the lowest formula weight among the members of the MXene family,^3,11−13^ which is promising for improving the energy density of supercapacitor and batteries.^10,14,15^ Ti_2_CT_*x*_ has also exhibited potential in photonic, photothermic, and optoelectronic applications.^16−19^ Zhang et al. demonstrated the third-order optical nonlinearity of Ti_2_CT_*x*_.^18^ Due to the optical nonlinearity, Ti_2_CT_*x*_ has been used as the saturable absorber for Q-switching fiber lasers for near-infrared (NIR) pulse generation.^16,18,20−22^ Szuplewska et al. demonstrated the potential of Ti_2_CT_*x*_ for photothermal therapy treatment.^17^ Moreover, in photovoltaics, the performance of perovskite solar cells can be improved by adding Ti_2_CT_*x*_ quantum dots.^19^ Ti_2_CT_*x*_ has shown great potential in many photonic and optoelectronic related fields, but the fundamental energy dissipation mechanisms of electrons and the lattice of Ti_2_CT_*x*_ are still unclear.^23^

Pump–probe (PP) spectroscopy is commonly used to investigate the carrier relaxation dynamics in metallic MXenes, which follow three distinct time scales similar to that of metals.^23−29^ (1) After pulsed laser irradiation, photoexcited electrons acquire excess energy of a few electronvolts (∼100 times higher than thermal energy). Electron–electron (e–e) scattering happens in the femtosecond time scale, increasing the temperature of electron gas above thousands of kelven.^23,24,26,27,30^ (2) Hot electrons then transfer energy to the lattice via electron–phonon (e–ph) coupling on a picosecond time scale.^23,24,30^ (3) After the electrons and lattice had reached quasi-thermal equilibrium above the environmental temperature, the hot lattice dissipates the thermal energy to the environment within hundreds of picoseconds or longer.^31−33^ While this long-lived process has been observed in MXene films, previous experiments (<5–6 ns) were insufficient to fully capture the lattice cooling dynamics in the metallic MXene thin films.^30,34^

The mechanisms of hot carrier relaxation and heat dissipation are not known for Ti_2_CT_*x*_, which are crucial in energy conversion applications. In this study, we investigate the energy dissipation dynamics in Ti_2_CT_*x*_ and compare it to that of Ti_3_C_2_T_*x*_ using transient absorption (TA) spectroscopy covering the time window from subpicoseconds to microseconds. Our results suggest that lattice heating is the dominant effect behind the TA response in both MXenes, without any signature of electronic excited states. We observe that hot carrier cooling in Ti_2_CT_*x*_ MXene takes ∼2.6 ps, notably slower than that in Ti_3_C_2_T_*x*_ MXene (∼1.6 ps).^23,31^ We find the absence of a hot-phonon bottleneck during the hot carrier cooling process in a Ti_2_CT_*x*_ film even at a high excitation density, different excitation wavelengths, and low environmental temperatures. The PP dynamics on a nano- to microsecond time scale provides information about the dissipation of heat from MXene films to the environment. In both MXene films, the heat dissipation takes as long as hundreds of nanoseconds, scaling linearly with the film thickness. Using a one-dimensional (1D) thermal diffusion model, thermal diffusivity for the Ti_2_CT_*x*_ film is ∼0.06 mm^2^ s^–1^ and that for the Ti_3_C_2_T_*x*_ film is ∼0.02 mm^2^ s^–1^. Such low thermal transfer properties can be caused by inefficient interflake heat transfer. Our study unravels the energy dissipation dynamics in Ti_2_CT_*x*_ films, which is instrumental for the development of MXene-based devices and photothermal management.

The MXenes are synthesized from MAX phase powders using an in situ hydrofluoric acid route by adding lithium fluoride to hydrochloric acid (HF) followed by minimally intensive layer delamination (MILD).^35,36^ The details of the sample preparation are described in the Supporting Information.

Figure 1a shows the steady-state absorption spectra of Ti_2_CT_*x*_ and Ti_3_C_2_T_*x*_ thin films. The characteristic plasmonic resonant peaks around 500 and 780 nm are observed for Ti_2_CT_*x*_ and Ti_3_C_2_T_*x*_, respectively, in agreement with the literature.^3,37^

**Figure 1:**
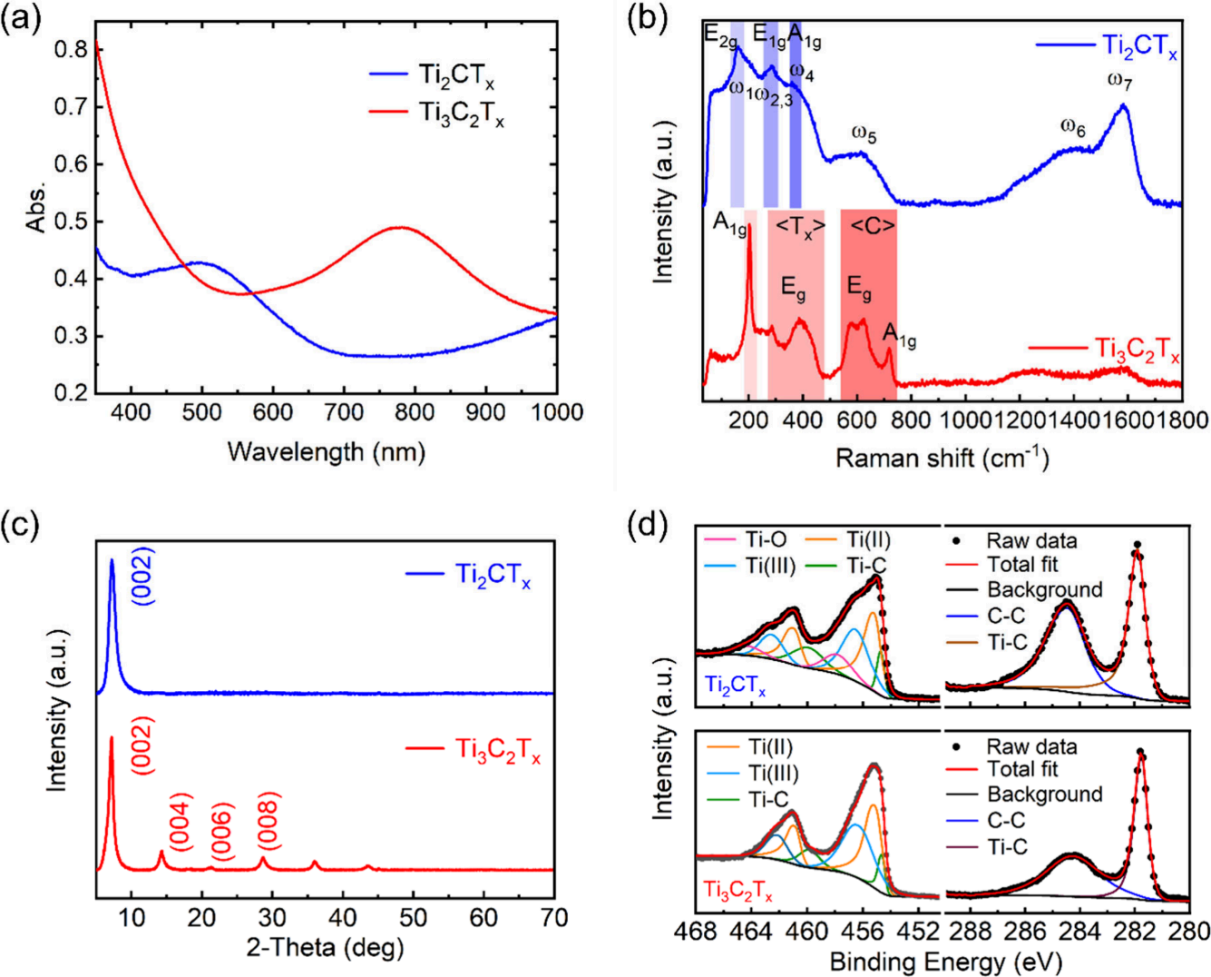
(a) Steady-state absorption spectra of Ti_2_CT_*x*_ and Ti_3_C_2_T_*x*_ films. (b) Raman spectra of Ti_2_CT_*x*_ and Ti_3_C_2_T_*x*_ films with 532 nm excitation. (c) XRD spectra of Ti_2_CT_*x*_ and Ti_3_C_2_T_*x*_ films. (d) High-resolution XPS spectra of Ti_2_CT_*x*_ and Ti_3_C_2_T_*x*_ films in the Ti 2p and C 1s regions.

Figure 1b shows the Raman spectra of Ti_2_CT_*x*_ and Ti_3_C_2_T_*x*_ films. The peaks (ω_1_, ω_2,3_, and ω_4_) of Ti_2_CT_*x*_ correlate to the E_2g_, E_1g_, and A_1g_ modes, respectively,^38^ which originate from the shear and longitudinal vibrations of the Ti and residual Al atoms.^9^ The upshifted and broadened peaks can be attributed to the removal of Al atoms, flake delamination, and termination. The ω_5_ peak, at ∼616 cm^–1^, may be attributed to nonstochiometric TiC_*x*_.^9^ The ω_6_ and ω_7_ peaks, observed at 1398 and 1578 cm^–1^, respectively, originate from the D and G bands of amorphous carbon, respectively.^39^ Two broad resonant regions of Ti_3_C_2_T_*x*_ (T_*x*_ between ∼223 and ∼480 cm^–1^ and C between ∼503 and ∼750 cm^–1^) originates from the in-plane E_g_ vibrations of T_*x*_ and the in-plane E_g_ vibrations plus the out-of-plane A_1g_ vibrations of the C atoms, respectively. An additional peak at ∼205 cm^–1^ originates from an out-of-plane A_1g_ vibrational mode of the carbon atoms, two Ti layers, and T_*x*_ (−OH, −O, and −F).^40,41^Figure S1 compares the Raman spectra between the MXenes and MAX phases.

The X-ray diffraction (XRD) patterns of Ti_2_CT_*x*_ and Ti_3_C_2_T_*x*_ films are shown in Figure 1c, and the observed peaks belong to basal-plane diffraction. The downshift of the first (002) peak (Ti_2_CT_*x*_ 2θ ∼ 7.2°, and Ti_3_C_2_T_*x*_ 2θ ∼ 7.2°) compared to those of Ti_2_AlC (2θ ∼ 13°) and Ti_3_AlC_2_ (2θ ∼ 10°) indicates the successful etching of the Al atoms and delamination of the MXenes (Figure S2).^42,43^

Figure 1d shows the X-ray photoelectron spectroscopy (XPS) spectra of Ti_2_CT_*x*_ and Ti_3_C_2_T_*x*_ films. The Ti 2p region comprises three doublet peaks for Ti_3_C_2_T_*x*_ and four doublet peaks for Ti_2_CT_*x*_.^8,41^ The Ti(II) and Ti(III) peaks are attributed to the T_*x*_ terminal groups.^40^ The asymmetric Ti–C (Ti 2p3, ∼454.7 eV; Ti 2p1, ∼460.0 eV) peaks are shifted to a higher binding energy compared with the MAX phase (Ti_3_AlC_2_, Ti–C 2p3, ∼454.6 eV),^44^ which is attributed to the etching of Al atoms. For Ti_2_CT_*x*_, the Ti–O (Ti 2p3, ∼458.0 eV; Ti 2p1, ∼464.4 eV) originates from oxidation of the Ti_2_CT_*x*_ at the edges forming −O and −OH terminations and TiO_2_ aggregates.^7,9^ This is not observed in the Ti 2p region for Ti_3_C_2_T_*x*_. Although both types of MXene are susceptible to oxidation when moisture or oxygen is present,^14,45,46^ all samples were stored under a N_2_ atmosphere before measurements. Compared with Ti_3_C_2_T_*x*_, Ti_2_CT_*x*_ is more sensitive to oxidation and might have already been oxidized during sample preparation.

The thickness (⟨*t*⟩) and lateral size (⟨*S*⟩) statistics of Ti_2_CT_*x*_ flakes (⟨*t*_Ti_2_CT_*x*__⟩ and ⟨*S*_Ti_2_CT_*x*__⟩) and Ti_3_C_2_T_*x*_ flakes (⟨*t*_Ti_3_C_2_T_*x*__⟩ and ⟨*S*_Ti_3_C_2_T_*x*__⟩) are characterized by atomic force microscopy (AFM). Figure S3 shows that ⟨*t*_Ti_2_CT_*x*__⟩ and ⟨*t*_Ti_3_C_2_T_*x*__⟩ both follow a log-normal distribution, peaking at ∼3.5 and ∼2.6 nm, respectively, and ⟨*S*_Ti_2_CT_*x*__⟩ and ⟨*S*_Ti_3_C_2_T_*x*__⟩ also follow a log-normal distribution, with peaks at ∼3.5 and ∼2.8 μm, respectively.

We compare the dynamics following photoexcitation of Ti_2_CT_*x*_ and Ti_3_C_2_T_*x*_ using TA spectroscopy with a time resolution of ∼100 fs. The MXene samples are excited by the pump pulse, and the change in absorbance (Δ*A*) induced by the pump pulse is recorded by the broadband probe pulse up to 6 ns.

Figures 2, 3, and S4 summarize the TA spectra and dynamics of Ti_2_CT_*x*_ and Ti_3_C_2_T_*x*_. In both MXenes, within the first ∼300 fs, the TA spectra deviate significantly from the spectra at later times [>10 ps (Figure 2 and Figure S5)]. The photophysical process within this time range is commonly attributed to e–e scattering.^23,24,30^ Due to the limit of the pulse duration (∼100 fs), the details of e–e scattering cannot be resolved in our study. Within the next 5 ps, the TA spectra of both MXenes (Figures S5 and S6) keep evolving, showing slight differences compared with those at later times (shown in Figure S6). We attribute the picosecond-level spectral shape changes to the hot carriers reaching thermal equilibrium with the lattice. The TA spectra of studied MXenes do not change after 10 ps and are attributed to the lattice heating effect when the carriers and lattice are already in thermal equilibrium.

**Figure 2:**
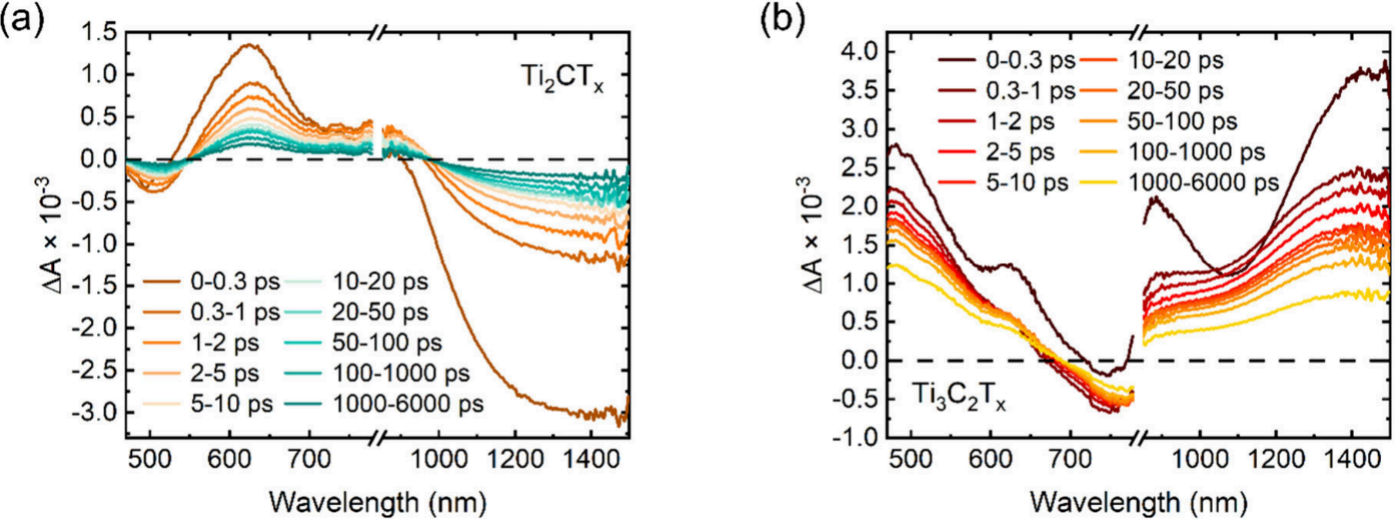
Visible–near-infrared TA spectra of (a) Ti_2_CT_*x*_ and (b) Ti_3_C_2_T_*x*_ films under 450 nm excitation with a fluence of 200 μJ cm^–2^.

**Figure 3:**
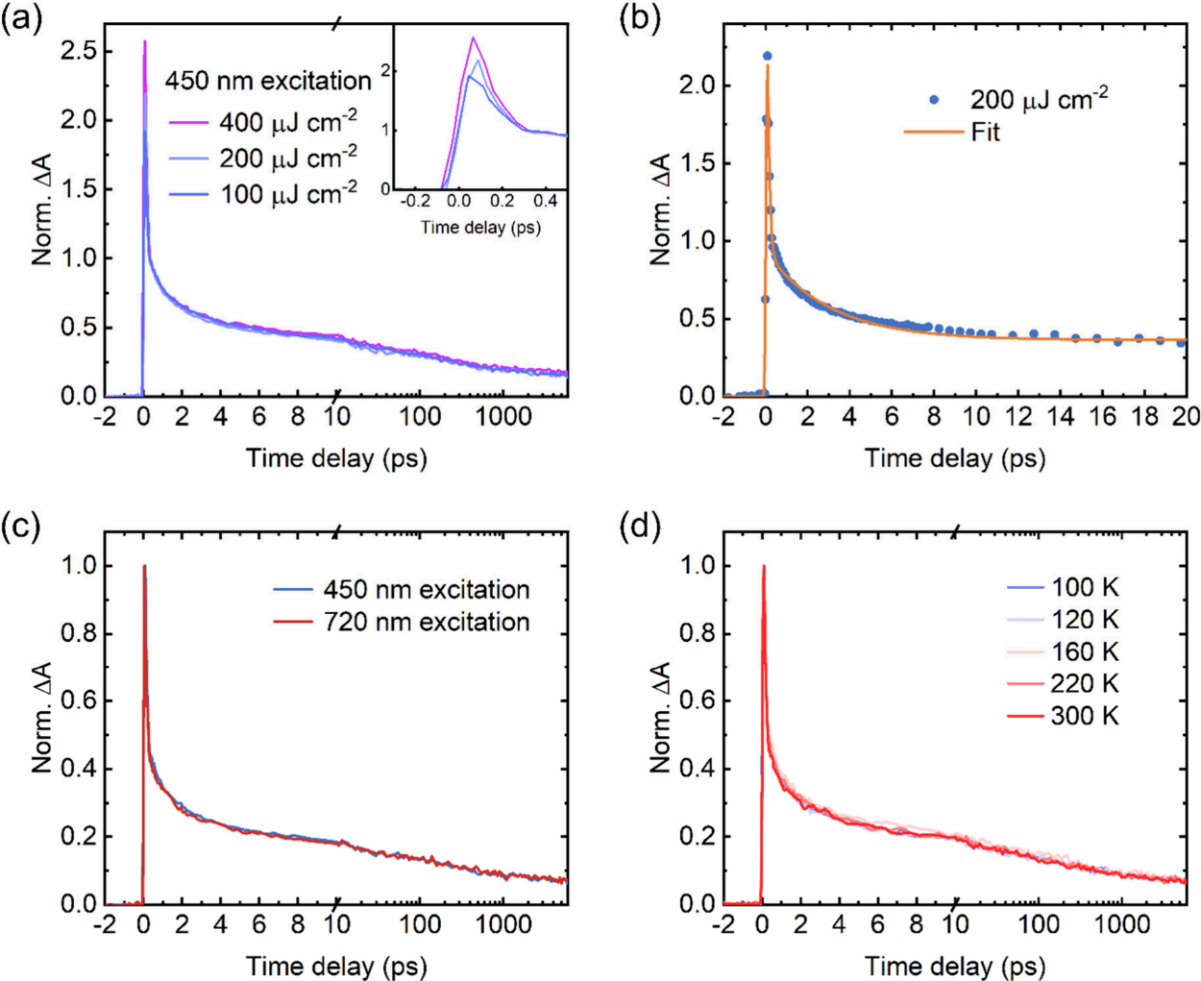
Ultrafast dynamics of Ti_2_CT*_x_* films. The dynamics probed at 630 nm is selected. (a) Kinetics of the Ti_2_CT_*x*_ film excited at 450 nm at different fluences normalized at 0.3 ps. (b) Fit of the kinetics excited at 450 nm (200 μJ cm^–2^) by eq 1. (c) Comparison of the kinetics between excitation at 450 nm (200 μJ cm^–2^, blue) and 720 nm (350 μJ cm^–2^, red). (d) Normalized kinetics of the Ti_2_CT_*x*_ film excited at 450 nm (200 μJ cm^–2^) at different environmental temperatures.

Figure 2 compares the TA spectra of Ti_2_CT_*x*_ and Ti_3_C_2_T_*x*_ excited at 450 nm. The negative photobleaching (PB) signal is observed in the areas corresponding to their plasmonic resonances shown in Figure 1a: ∼500 nm in Ti_2_CT_*x*_ and ∼780 nm in Ti_3_C_2_T_*x*_. Similar PB spectral features have been observed in previous studies.^30,47^ Positive photoinduced absorption (PIA) spectral features can be observed on the sides of the PB signal for both MXenes. In the NIR region (850–1500 nm), another PB signal with a higher intensity is observed in Ti_2_CT_*x*_ from 1000 to >1500 nm; however, only the PIA signal is observed in Ti_3_C_2_T_*x*_. The PB signal for Ti_2_CT_*x*_ MXene in the NIR region can be assigned as the resonance of a high concentration of plasma.^3^

Figure 3 presents the subpicosecond to nanosecond dynamics of the Ti_2_CT_*x*_ film under different experimental conditions. According to the spectral shape analysis presented above, all of the probe wavelengths demonstrate identical kinetic behavior. We select the Δ*A* signal probed at 630 nm for Ti_2_CT_*x*_ and 500 nm for Ti_3_C_2_T_*x*_ to represent the cooling dynamics in the studied films, as these wavelengths provide the best signal:noise ratio. These selected probing wavelengths are consistently used in subsequent pump–probe measurements, extending up to the microsecond time scale. Figure 3a shows the dynamics of the Ti_2_CT_*x*_ film excited at 450 nm at different excitation fluences. The e–e scattering shows the dependence on the fluence in Ti_2_CT_*x*_, and therefore, the dynamics is normalized at 0.3 ps.

As shown in Figure 3a, after 0.3 ps, the Δ*A* signal of Ti_2_CT_*x*_ shows two decaying components: one on a picosecond time scale and the other on a nanosecond time scale. The picosecond-level decay is attributed to hot carrier cooling, achieving thermal equilibrium with the material lattice.^23^ The subsequent decay of the Δ*A* amplitude represents the heat dissipation from hot lattice to environment on the nano- to microsecond time scale.^30^Figure 3a shows that the TA signal after 0.3 ps is independent of laser fluence, indicating that carrier cooling in the Ti_2_CT_*x*_ film is unaffected by the excitation density.

To obtain hot carrier cooling time, Δ*A* dynamics within the first 20 ps is fitted by a biexponential decay plus a baseline, convoluted with the pulse duration, *G*(*t*) (∼100 fs):
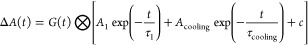
1where *A*_1_, τ_1_, *A*_cooling_, and τ_cooling_ represent the amplitudes and lifetimes of the fast-decaying component and carrier cooling, respectively, and *c* is a constant value to estimate the long-lived component. Figure 3b shows the fitted curve, and the carrier cooling time of Ti_2_CT_*x*_ is 2.6 ± 0.1 ps, which is longer than that of Ti_3_C_2_T_*x*_ [1.6 ± 0.2 ps (Figure S4)]. Apart from differences in e–ph coupling between the two MXene films, the density of free carriers and defects can also contribute to the observed variations.^48−50^ Furthermore, as shown in panels c and d of Figure 3, the cooling dynamics of Ti_2_CT_*x*_ is not affected by the excitation wavelength or temperature, similar to what has been reported for Ti_3_C_2_T_*x*_ in the Supporting Information and by Zhao et al.^51^ These results highlight that hot carrier cooling in Ti_2_CT_*x*_ is not affected by the hot-phonon bottleneck effect that is widely observed in graphene and transition metal dichalcogenides (TMDs).^50,52−54^ In graphene and TMDs, hot carrier relaxation slows at higher densities due to a lack of longitudinal optical (LO) phonons. The absence of the hot-phonon bottleneck effect has been extensively found in the MXene family.^23,30,47,55−57^ This can be attributed to the similar e–ph coupling constants in different MXenes^25^ and a high free electron density in metallic MXenes that facilitates hot carrier relaxation via carrier–carrier interactions.^48−50^ The absence of the hot-phonon bottleneck in MXenes suggests broad potential for application, especially in cryogenic and high-power environments.

As shown in panels b and f of Figure S7, the TA spectral shape at 100 ps of both MXenes can be mostly described by the thermally induced absorption change. Therefore, we assign the TA response after 10 ps, when carrier cooling is complete, to the thermochromic phenomena. The increase in the temperature of electron gas and lattice after photoexcitation leads to the modulation of absorption and transmission properties. To gain insight into the origin of the thermochromic effect, we compare TA spectra to the first and second derivatives of the respective absorption spectra (Figure S8). The line shape of the TA spectrum of Ti_2_CT_*x*_ at 100 ps can be described well as a linear combination of the first and second derivatives  of the steady-state absorption spectrum (Figure 4a). Therefore, the steady-state absorption spectrum of Ti_2_CT_*x*_ MXene is shifted to red and broadened after excitation. As shown in Figure 4b, the line shape of the TA spectrum of Ti_3_C_2_T_*x*_ at 100 ps can be represented solely by the inverse of the first derivative  of the steady-state absorption spectrum, without the need for the second derivative, which is consistent with the literature.^23^ Analogous to the Stark effect,^58,59^ the thermochromic behavior of the studied MXenes suggests that the surface dipole moment of Ti_2_CT_*x*_ is more temperature-sensitive than that of Ti_3_C_2_T_*x*_. We attribute this to differences in the density and types of surface functional groups on the two samples.^60,61^ The same spectral shift of the plasmonic resonant peaks in both MXenes is observed in the steady state at different temperatures, as shown in panels c, d, g, and h of Figure S7. The TA spectral line shape analysis of Ti_2_CT_*x*_ and Ti_3_C_2_T_*x*_ indicates that the primary process is the thermochromic effect with no evidence of excited-state dynamics.

**Figure 4:**
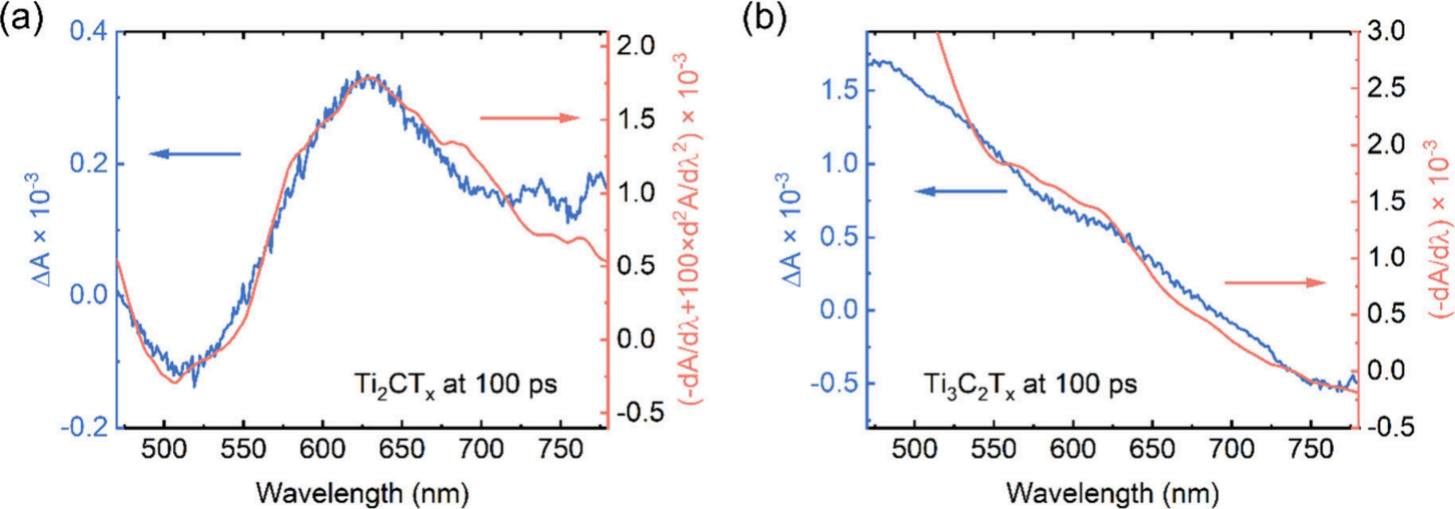
(a) TA spectrum of the Ti_2_CT_*x*_ film at 100 ps compared to the linear combination of the first and second derivatives of the steady-state absorption spectrum. (b) TA spectrum of the Ti_3_C_2_T_*x*_ film at 100 ps compared to the first derivative of the steady-state absorption spectrum.

As shown in Figure 3 and Figure S4, both MXene films exhibit initial dynamics followed by a slower process with a lifetime of >6 ns, attributed to heat dissipation from the hot lattice to the environment (i.e., lattice cooling).^23,30^ While Li et al. demonstrated faster lattice cooling for Ti_3_C_2_T_*x*_ flakes in a liquid suspension on the picosecond time scale, the lattice cooling dynamics of MXene films have not been previously studied.^31,32^ To explore this, we conducted PP experiments on time scales from nanoseconds to microseconds.

Figure 5 summarizes the lattice cooling dynamics of Ti_2_CT_*x*_ and Ti_3_C_2_T_*x*_ films. Heat flow in MXene films was measured by exciting them at 1064 nm (pulse duration of ∼15 ns) and then probing the thermochromic effect at ∼630 and ∼500 nm for the Ti_2_CT_*x*_ and Ti_3_C_2_T_*x*_ films, respectively. Here we record the change in the transmittance of the probe beam (Δ*T*/*T*) as a function of the delay time. As shown in Figure S9, the lattice cooling dynamics is independent of the excitation density confirming that only heat distribution effects are present in the system. We then investigate the lattice cooling dynamics in both MXene films as a function of the film thickness, as shown in panels a and b of Figure 5. The lattice cooling dynamics of Ti_2_CT_*x*_ (Figure 5a) and Ti_3_C_2_T_*x*_ (Figure 5b) films are fitted by a monoexponential decay, , and a biexponential decay, , respectively, convoluted with the pump pulse duration. The time constant of the monoexponential fit is used as the lattice cooling time of Ti_2_CT_*x*_ films, while the lattice heat dissipation time of Ti_3_C_2_T_*x*_ films is the average of the two decaying components:^33^
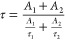
2

**Figure 5:**
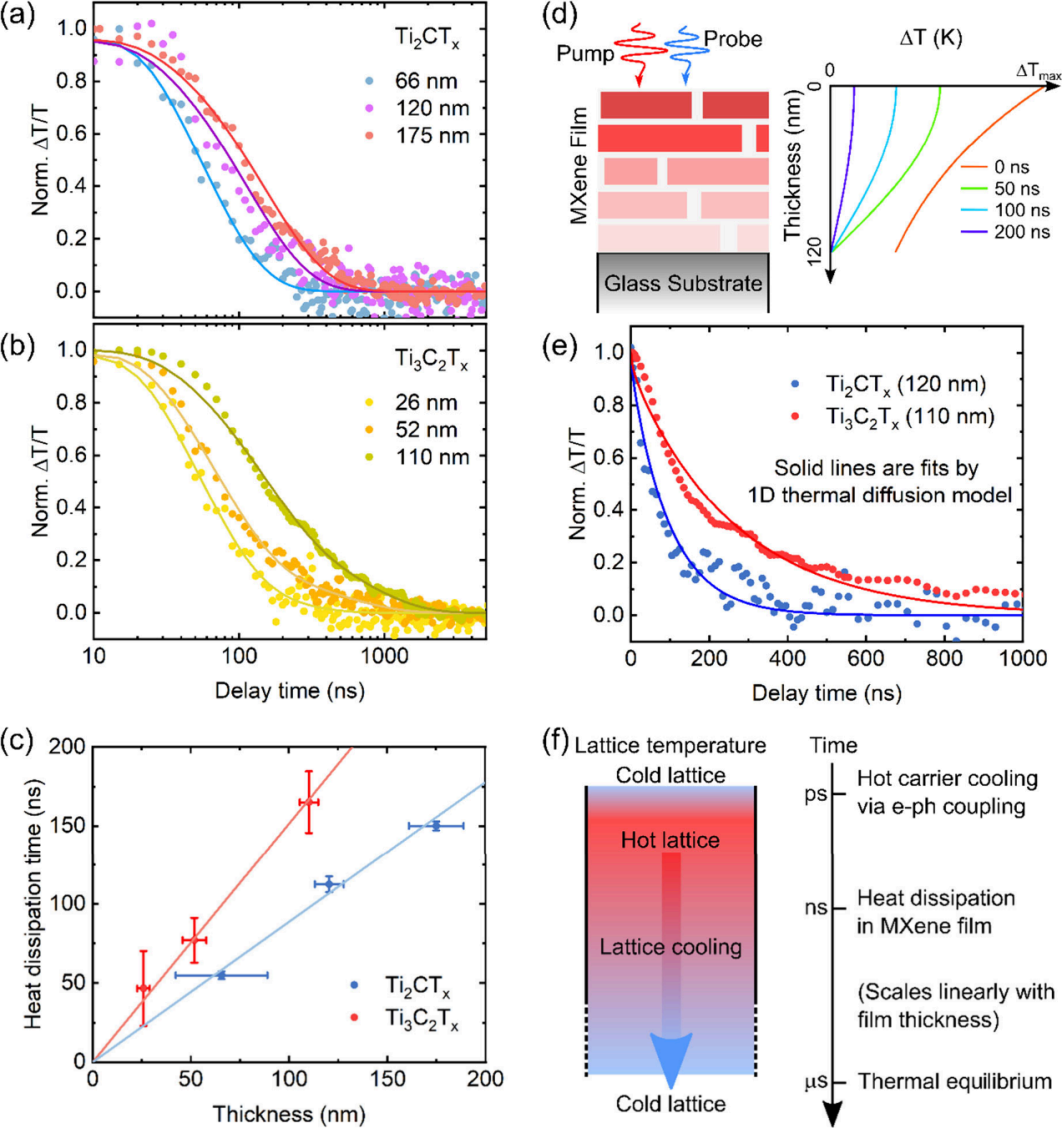
Lattice cooling dynamics of (a) Ti_2_CT_*x*_ films and (b) Ti_3_C_2_T_*x*_ films at different thicknesses with 1064 nm excitation (2.8 mJ cm^–2^ for Ti_2_CT_*x*_ MXene films and 1.8 mJ cm^–2^ for Ti_3_C_2_T_*x*_ MXene films). (c) Lattice cooling times in Ti_2_CT_*x*_ and Ti_3_C_2_T_*x*_ MXene films as a function of film thickness. The solid lines are the linear fits with a fixed intercept at zero. The error bar for film thickness is the standard deviation of the thickness measured at several points on an individual sample by a profilometer. (d) Scheme of the 1D thermal diffusion model of heat dissipation in the MXene flake-based film on the substrate. The plot shows the heat distribution within the Ti_2_CT_*x*_ film at different time delays. (e) Fits of PP dynamics of Ti_2_CT_*x*_ (120 nm, blue) and Ti_3_C_2_T_*x*_ (110 nm, red) films with the 1D thermal diffusion model. (f) Energy dissipation time scales in the MXene film from carrier cooling to lattice cooling.

Figure 5c shows that the heat dissipation time in both MXene films increases linearly with the film thickness. Figure 5c also shows that at a constant film thickness, the heat dissipation time in the Ti_2_CT_*x*_ film is faster than that in the Ti_3_C_2_T_*x*_ film.

To obtain the thermal diffusivity (α) in the MXene films, we use a classical 1D thermal diffusion model to fit the PP dynamics in panels a and b of Figure 5 (details in the Supporting Information). According to our previous studies of Ti_3_C_2_T_*x*_ flakes, the micrometer-sized MXene flakes prefer to stack almost parallelly to the substrate.^41^Figure 5d illustrates the simulation of the 1D heat flow across the thickness of the MXene flake network. Figure 5e shows that the lattice cooling dynamics in the Ti_2_CT_*x*_ MXene film (120 nm) and Ti_3_C_2_T_*x*_ MXene film (110 nm) can be described well by the model. The fitted α for the Ti_2_CT_*x*_ film is ∼0.06 mm^2^ s^–1^, and the α for the Ti_3_C_2_T_*x*_ film is ∼0.02 mm^2^ s^–1^ with errors of <5%, which are orders of magnitude lower than typical metals.^62^ The relatively higher thermal diffusivity of the Ti_2_CT_*x*_ film may be due to unavoidable oxidation, as shown in the XPS results in Figure 1d, which is suggested to improve heat transfer by reducing the gap between flakes.^63^ According to the Wiedemann–Franz law, the thermal diffusivity is expected to scale linearly with electrical conductivity. However, the thermal diffusivity for the two metallic MXenes deviates from the Wiedemann–Franz law. The deviation from the Wiedemann–Franz law can be caused by the strong e–ph scattering in MXenes, which can reduce the thermal conductivity in these 2D metallic materials.^64^

With regard to materials, the low thermal diffusivity of MXene films can be explained by slow interflake heat transfer. As illustrated in Figure 5d, the heat from the upper flakes will propagate across multiple flakes to the substrate and be dissipated. The slow interflake heat transfer can be caused by the packing of the flakes and the surface functional groups (such as −OH, =O, −Cl, and −F).^63,65^ The full energy dissipation time scales of Ti_2_CT_*x*_ and Ti_3_C_2_T_*x*_ films are summarized in Figure 5f.

In summary, we successfully synthesize Ti_2_CT_*x*_ and Ti_3_C_2_T_*x*_ MXenes using in situ HF selective etching and the MILD method, confirmed by Raman spectroscopy, XRD, and XPS. We investigate the energy dissipation dynamics in Ti_2_CT_*x*_ films compared to widely studied Ti_3_C_2_T_*x*_ films, from femtoseconds to microseconds. The carrier cooling time in Ti_2_CT_*x*_ (∼2.6 ps) is 1 ps longer than that in Ti_3_C_2_T_*x*_, with no evidence of a hot-phonon bottleneck. The main features of TA spectra of the Ti_2_CT_*x*_ film can be solely described by the thermochromic effect. However, in both MXene films, the dissipation of heat from the lattice to the environment takes as long as hundreds of nanoseconds, scaling linearly with the film thickness. Using a 1D thermal diffusion model, we extract the thermal diffusivities of the Ti_2_CT_*x*_ film (∼0.06 mm^2^ s^–1^) and Ti_3_C_2_T_*x*_ film (∼0.02 mm^2^ s^–1^). Such low thermal diffusivities can be caused by inefficient interflake heat transfer. Our study unravels the energy dissipation mechanisms in Ti_2_CT_*x*_ thin films, which is relevant for both fundamental studies and applications.

## Methods

The steady-state absorption spectrum was measured with a Cary 60 UV–vis spectrophotometer (Agilent). In the temperature-dependent measurement, the samples were measured within the cryostat (OptistatDN-V, Oxford Instruments) under high vacuum. The temperature was controlled by a temperature controller (MercuryiTC). Raman spectra were acquired with a Renishaw inVia micro-Raman spectrometer (WiRe 4.1) using a 532 nm laser with a 50× objective lens. An incident power of ≤1 mW was used to avoid thermal damage. Samples for Raman spectroscopy were deposited as thin films on pre-cleaned (isopropanol/acetone) Si/SiO_2_ substrates (Si-Mat). XPS spectra were acquired with a Kratos Axis Supra instrument, using an Al α X-ray source focused into an 800 μm spot. The XPS samples were deposited as thin films on precleaned glass. The data were analyzed with Avantage 5.99 (Thermo Scientific). XRD patterns were acquired by a Bruker diffractometer (D8 advance, AXS system), scanning within a 2θ range from 5° to 70° at a step scan of 0.02°, with a fixed slit size of 0.6 mm. The XRD samples were deposited as thin films on precleaned glass. A profilometer (Dektak 150, Veeco) was utilized to estimate the thickness of the as-prepared MXene films using a stylus (radius of 5 μm) at a scanning force of 5 mg. For each MXene film, the measurements were collected from three different regions, and the mean thickness and its standard deviation were extracted. All of the films were carefully stored in a N_2_ atmosphere before all measurements to avoid fast oxidation.^7^ During all time-resolved measurements at room temperature, all samples were sealed in a N_2_-purged cuvette. Details for AFM characterization, TA spectroscopy, and nanosecond-to-microsecond pump–probe spectroscopy are available in the Supporting Information.

## Supporting Information Available

Details for AFM statistics of MXene flake size (lateral size and thickness), XRD patterns of MXene compared to that of the MAX phase, Raman spectra of MXene compared to those of the MAX phase, details of TA spectroscopy and nanosecond-to-microsecond-PP spectroscopy, first and second derivatives of the steady-state absorption spectra of Ti_2_CT_*x*_ and Ti_3_C_2_T_*x*_ MXene films at room temperature, steady-state absorption spectra of Ti_2_CT_*x*_ and Ti_3_C_2_T_*x*_ MXene films at different temperatures and line shape analysis, ultrafast dynamics of the Ti_3_C_2_T_*x*_ MXene film, nanosecond-to-microsecond-PP dynamics of Ti_2_CT_*x*_ and Ti_3_C_2_T_*x*_ MXene films with 1064 nm excitation at different fluences, and details of the 1D thermal diffusion model (PDF)

## References

[ref1] Naguib M.; Mochalin V. N.; Barsoum M. W.; Gogotsi Y. 25th Anniversary Article: MXenes: A New Family of Two-Dimensional Materials. Adv. Mater. 2014, 26, 992–1005. 10.1002/adma.201304138. 24357390

[ref2] VahidMohammadi A.; Rosen J.; Gogotsi Y. The World of Two-Dimensional Carbides and Nitrides (MXenes). Science 2021, 372, eabf1581 10.1126/science.abf1581. 34112665

[ref3] Maleski K.; Shuck C. E.; Fafarman A. T.; Gogotsi Y. The Broad Chromatic Range of Two-Dimensional Transition Metal Carbides. Adv. Opt. Mater. 2021, 9, 2001563 10.1002/adom.202001563.

[ref4] Zhu Z.; Zou Y.; Hu W.; Li Y.; Gu Y.; Cao B.; Guo N.; Wang L.; Song J.; Zhang S.; Gu H.; Zeng H. Near-Infrared Plasmonic 2D Semimetals for Applications in Communication and Biology. Adv. Funct Mater. 2016, 26, 1793–1802. 10.1002/adfm.201504884.

[ref5] Velusamy D. B.; El-Demellawi J. K.; El-Zohry A. M.; Giugni A.; Lopatin S.; Hedhili M. N.; Mansour A. E.; Fabrizio E. Di; Mohammed O. F.; Alshareef H. N. MXenes for Plasmonic Photodetection. Adv. Mater. 2019, 31, 1807658 10.1002/adma.201807658. 31222823

[ref6] Xie X.; Zhang N. Positioning MXenes in the Photocatalysis Landscape: Competitiveness, Challenges, and Future Perspectives. Adv. Funct. Mater. 2020, 30, 2002528 10.1002/adfm.202002528.

[ref7] Roy C.; De S. K.; Banerjee P.; Pradhan S.; Bhattacharyya S. Investigating Suitable Medium for the Long-Duration Storage of Ti_2_CT_x_ MXene. J. Alloys Compd. 2023, 938, 168471 10.1016/j.jallcom.2022.168471.

[ref8] Liu S.; Liu J.; Liu X.; Shang J.; Xu L.; Yu R.; Shui J. Hydrogen Storage in Incompletely Etched Multilayer Ti_2_CT_x_ at Room Temperature. Nat. Nanotechnol 2021, 16, 331–336. 10.1038/s41565-020-00818-8. 33398176

[ref9] Melchior S. A.; Raju K.; Ike I. S.; Erasmus R. M.; Kabongo G.; Sigalas I.; Iyuke S. E.; Ozoemena K. I. High-Voltage Symmetric Supercapacitor Based on 2D Titanium Carbide (MXene, Ti_2_CT_x_)/Carbon Nanosphere Composites in a Neutral Aqueous Electrolyte. J. Electrochem. Soc. 2018, 165, A501–A511. 10.1149/2.0401803jes.

[ref10] Guan Y.; Zhao R.; Cong Y.; Chen K.; Wu J.; Zhu H.; Dong Z.; Zhang Q.; Yuan G.; Li Y.; Zhang J.; Li X. Flexible Ti_2_C MXene Film: Synthesis, Electrochemical Performance and Capacitance Behavior. Chemical Engineering Journal 2022, 433, 133582 10.1016/j.cej.2021.133582.

[ref11] Ying G.; Dillon A. D.; Fafarman A. T.; Barsoum M. W. Transparent, Conductive Solution Processed Spincast 2D Ti_2_CT_x_ (MXene) Films. Mater. Res. Lett. 2017, 5, 391–398. 10.1080/21663831.2017.1296043.

[ref12] Halim J.; Persson I.; Moon E. J.; Kühne P.; Darakchieva V.; Persson P. O. Å.; Eklund P.; Rosen J.; Barsoum M. W. Electronic and Optical Characterization of 2D Ti_2_C and Nb_2_C (MXene) Thin Films. J. Phys.: Condens. Matter 2019, 31, 165301 10.1088/1361-648X/ab00a2. 30669136

[ref13] Yorulmaz U.; Özden A.; Perkgöz N. K.; Ay F.; Sevik C. Vibrational and Mechanical Properties of Single Layer MXene Structures: A First-Principles Investigation. Nanotechnology 2016, 27, 335702 10.1088/0957-4484/27/33/335702. 27377143

[ref14] Ahmed B.; Anjum D. H.; Hedhili M. N.; Gogotsi Y.; Alshareef H. N. H_2_O_2_ Assisted Room Temperature Oxidation of Ti_2_C MXene for Li-Ion Battery Anodes. Nanoscale 2016, 8, 7580–7587. 10.1039/C6NR00002A. 26984324

[ref15] Rakhi R. B.; Ahmed B.; Hedhili M. N.; Anjum D. H.; Alshareef H. N. Effect of Postetch Annealing Gas Composition on the Structural and Electrochemical Properties of Ti_2_CT_x_ MXene Electrodes for Supercapacitor Applications. Chem. Mater. 2015, 27, 5314–5323. 10.1021/acs.chemmater.5b01623.

[ref16] Ahmad H.; Kamely A. A.; Samion M. Z.; Soo Y. H. Ti2C MXene for Multi-Wavelength Enhancement in S-Band Q-Switched Thulium Doped Fluoride Fiber Laser. Optical Fiber Technology 2022, 68, 102790 10.1016/j.yofte.2021.102790.

[ref17] Szuplewska A.; Kulpińska D.; Dybko A.; Jastrzębska A. M.; Wojciechowski T.; Rozmysłowska A.; Chudy M.; Grabowska-Jadach I.; Ziemkowska W.; Brzózka Z.; Olszyna A. 2D Ti_2_C (MXene) as a Novel Highly Efficient and Selective Agent for Photothermal Therapy. Materials Science and Engineering: C 2019, 98, 874–886. 10.1016/j.msec.2019.01.021. 30813093

[ref18] Zhang T.; Chu H.; Li Y.; Zhao S.; Ma X.; Pan H.; Li D. Third-Order Optical Nonlinearity in Ti_2_C MXene for Q-Switching Operation at 1–2 μm. Opt Mater. (Amst) 2022, 124, 112054 10.1016/j.optmat.2022.112054.

[ref19] Luo Z.; Liu Y.; Zhang X.; Wang C.; Wen S.; Zhang W.; Chen S.; Zheng W. Grain Boundary Engineering with Cl-Terminated Ti_2_C Quantum Dots for Enhancing Perovskite Solar Cell Performance. ACS Sustain Chem. Eng. 2023, 11, 7072–7082. 10.1021/acssuschemeng.2c07754.

[ref20] Ahmad H.; Abdul Kahar N. H.; Ramli R.; Yusoff N.; Reduan S. A.; Ismail M. F.; Lim K. S.; Chong W. Y.; Yasin M. The Performance of Ti_2_C MXene and Ti2AlC MAX Phase as Saturable Absorbers for Passively Mode-Locked Fiber Laser. Optical Fiber Technology 2021, 67, 102683 10.1016/j.yofte.2021.102683.

[ref21] Ahmad H.; Jasmine Mohd Makhfuz M.; Yusoff N.; Zakaria R. The Effect of Ti_2_C MXene on the Performance of Optical Fiber-Based Surface Plasmon Resonance Sensor towards Lead Detection. Materials Science and Engineering: B 2024, 302, 117232 10.1016/j.mseb.2024.117232.

[ref22] Ahmad H.; Mohd Makhfuz M. J.; Yusoff N.; Azam A. D.; Samion M. Z. Synthesis of 2D Titanium Carbide Ti_2_C, Its Characteristics, and Nonlinear Optical Properties. Opt Mater. (Amst) 2023, 135, 113340 10.1016/j.optmat.2022.113340.

[ref23] Zhang Q.; Yan L.; Yang M.; Wu G.; Hu M.; Li J.; Yuan K.; Yang X. Ultrafast Transient Spectra and Dynamics of MXene (Ti_3_C_2_T_x_) in Response to Light Excitations of Various Wavelengths. J. Phys. Chem. C 2020, 124, 6441–6447. 10.1021/acs.jpcc.9b11652.

[ref24] Li G.; Kushnir K.; Dong Y.; Chertopalov S.; Rao A. M.; Mochalin V. N.; Podila R.; Titova L. V. Equilibrium and Non-Equilibrium Free Carrier Dynamics in 2D Ti_3_C_2_T_x_ MXenes: THz Spectroscopy Study. 2d Mater. 2018, 5, 035043 10.1088/2053-1583/aacb9e.

[ref25] Zheng W.; Sun B.; Li D.; Gali S. M.; Zhang H.; Fu S.; Di Virgilio L.; Li Z.; Yang S.; Zhou S.; Beljonne D.; Yu M.; Feng X.; Wang H. I.; Bonn M. Band Transport by Large Fröhlich Polarons in MXenes. Nat. Phys. 2022, 18, 544–550. 10.1038/s41567-022-01541-y.

[ref26] Chen J. K.; Tzou D. Y.; Beraun J. E. A Semiclassical Two-Temperature Model for Ultrafast Laser Heating. Int. J. Heat Mass Transf 2006, 49, 307–316. 10.1016/j.ijheatmasstransfer.2005.06.022.

[ref27] Jiang L.; Tsai H. L. Improved Two-Temperature Model and Its Application in Ultrashort Laser Heating of Metal Films. J. Heat Transfer 2005, 127, 1167–1173. 10.1115/1.2035113.

[ref28] Zhang X.; Huang C.; Wang M.; Huang P.; He X.; Wei Z. Transient Localized Surface Plasmon Induced by Femtosecond Interband Excitation in Gold Nanoparticles. Sci. Rep. 2018, 8, 10499 10.1038/s41598-018-28909-6. 30002475 PMC6043523

[ref29] Ahmadi T. S.; Logunov S. L.; El-Sayed M. A. Picosecond Dynamics of Colloidal Gold Nanoparticles. J. Phys. Chem. 1996, 100, 8053–8056. 10.1021/jp960484e.

[ref30] Colin-Ulloa E.; Fitzgerald A.; Montazeri K.; Mann J.; Natu V.; Ngo K.; Uzarski J.; Barsoum M. W.; Titova L. V. Ultrafast Spectroscopy of Plasmons and Free Carriers in 2D MXenes. Adv. Mater. 2023, 35, 2208659 10.1002/adma.202208659. 36369973

[ref31] Li J.; Chi Z.; Qin R.; Yan L.; Lin X.; Hu M.; Shan G.; Chen H.; Weng Y. X. Hydrogen Bond Interaction Promotes Flash Energy Transport at MXene-Solvent Interface. J. Phys. Chem. C 2020, 124, 10306–10314. 10.1021/acs.jpcc.0c01039.

[ref32] Li J.; Zhang Q.; Yan L.; Wu G.; Hu M.; Lin X.; Yuan K.; Yang X. Ultrafast Flash Energy Conductance at MXene-Surfactant Interface and Its Molecular Origins. Adv. Mater. Interfaces 2019, 6, 1901461 10.1002/admi.201901461.

[ref33] Hu M.; Wang X.; Hartland G. V.; Salgueiriño-Maceira V.; Liz-Marzán L. M. Heat Dissipation in Gold-Silica Core-Shell Nanoparticles. Chem. Phys. Lett. 2003, 372, 767–772. 10.1016/S0009-2614(03)00506-2.

[ref34] Rawat A.; Chourasia N. K.; Saini S. K.; Rajput G.; Yadav A.; Chourasia R. K.; Gupta G.; Kulriya P. K. Investigation of Charge Carrier Dynamics in a Ti_3_C_2_T_x_ MXene for Ultrafast Photonics Applications. Mater. Adv. 2023, 4, 6427–6438. 10.1039/D3MA00429E.

[ref35] Ghidiu M.; Lukatskaya M. R.; Zhao M. Q.; Gogotsi Y.; Barsoum M. W. Conductive Two-Dimensional Titanium Carbide “clay” with High Volumetric Capacitance. Nature 2014, 516, 78–81. 10.1038/nature13970. 25470044

[ref36] Alhabeb M.; Maleski K.; Anasori B.; Lelyukh P.; Clark L.; Sin S.; Gogotsi Y. Guidelines for Synthesis and Processing of Two-Dimensional Titanium Carbide (Ti_3_C_2_T_x_ MXene). Chem. Mater. 2017, 29, 7633–7644. 10.1021/acs.chemmater.7b02847.

[ref37] Li Y.; Xiong C.; Huang H.; Peng X.; Mei D.; Li M.; Liu G.; Wu M.; Zhao T.; Huang B. 2D Ti_3_C_2_T_x_ MXenes: Visible Black but Infrared White Materials. Adv. Mater. 2021, 33, 2103054 10.1002/adma.202103054. 34463370

[ref38] Presser V.; Naguib M.; Chaput L.; Togo A.; Hug G.; Barsoum M. W. First-order Raman Scattering of the MAX Phases: Ti_2_AlN, Ti_2_AlC_0.5_N_0.5_, Ti_2_AlC, (Ti_0.5_V_0.5_) _2_ AlC, V_2_AlC, Ti_3_AlC_2_, and Ti_3_GeC_2_. J. Raman Spectrosc. 2012, 43, 168–172. 10.1002/jrs.3036.

[ref39] Lai S.; Jeon J.; Jang S. K.; Xu J.; Choi Y. J.; Park J.-H.; Hwang E.; Lee S. Surface Group Modification and Carrier Transport Properties of Layered Transition Metal Carbides (Ti_2_CT_x_, T: – OH, – F and – O). Nanoscale 2015, 7, 19390–19396. 10.1039/C5NR06513E. 26535782

[ref40] Kang R.; Zhang Z.; Guo L.; Cui J.; Chen Y.; Hou X.; Wang B.; Lin C.-T.; Jiang N.; Yu J. Enhanced Thermal Conductivity of Epoxy Composites Filled with 2D Transition Metal Carbides (MXenes) with Ultralow Loading. Sci. Rep. 2019, 9, 9135 10.1038/s41598-019-45664-4. 31235757 PMC6591414

[ref41] Piatti E.; Arbab A.; Galanti F.; Carey T.; Anzi L.; Spurling D.; Roy A.; Zhussupbekova A.; Patel K. A.; Kim J. M.; Daghero D.; Sordan R.; Nicolosi V.; Gonnelli R. S.; Torrisi F. Charge Transport Mechanisms in Inkjet-Printed Thin-Film Transistors Based on Two-Dimensional Materials. Nat. Electron 2021, 4, 893–905. 10.1038/s41928-021-00684-9.

[ref42] Liu F.; Zhou A.; Chen J.; Zhang H.; Cao J.; Wang L.; Hu Q. Preparation and Methane Adsorption of Two-Dimensional Carbide Ti_2_C. Adsorption 2016, 22, 915–922. 10.1007/s10450-016-9795-8.

[ref43] Yong S.; Yao C.; Hillier N.; Kim H.; Holicky M.; Liu S.; Doherty R.; Torrisi F.; Beeby S. Ti_3_C_2_ MXene as Additive for Low-Cost Textile Supercapacitors with Enhanced Electrical Performance. Adv. Mater. Technol. 2024, 9, 2301266 10.1002/admt.202301266.

[ref44] Myhra S.; Crossley J. A. A.; Barsoum M. W. Crystal-Chemistry of the Ti_3_AlC_2_ and Ti_4_AlN_3_ Layered Carbide/Nitride Phases—Characterization by XPS. J. Phys. Chem. Solids 2001, 62, 811–817. 10.1016/S0022-3697(00)00268-7.

[ref45] Zhang C. J.; Pinilla S.; McEvoy N.; Cullen C. P.; Anasori B.; Long E.; Park S. H.; Seral-Ascaso A.; Shmeliov A.; Krishnan D.; Morant C.; Liu X.; Duesberg G. S.; Gogotsi Y.; Nicolosi V. Oxidation Stability of Colloidal Two-Dimensional Titanium Carbides (MXenes). Chem. Mater. 2017, 29, 4848–4856. 10.1021/acs.chemmater.7b00745.

[ref46] Seredych M.; Shuck C. E.; Pinto D.; Alhabeb M.; Precetti E.; Deysher G.; Anasori B.; Kurra N.; Gogotsi Y. High-Temperature Behavior and Surface Chemistry of Carbide MXenes Studied by Thermal Analysis. Chem. Mater. 2019, 31, 3324–3332. 10.1021/acs.chemmater.9b00397.

[ref47] Zhang Q.; Li J.; Wen J.; Li W.; Chen X.; Zhang Y.; Sun J.; Yan X.; Hu M.; Wu G.; Yuan K.; Guo H.; Yang X. Simultaneous Capturing Phonon and Electron Dynamics in MXenes. Nat. Commun. 2022, 13, 7900 10.1038/s41467-022-35605-7. 36550116 PMC9780317

[ref48] Hopper T. R.; Gorodetsky A.; Jeong A.; Krieg F.; Bodnarchuk M. I.; Maimaris M.; Chaplain M.; Macdonald T. J.; Huang X.; Lovrincic R.; Kovalenko M. V.; Bakulin A. A. Hot Carrier Dynamics in Perovskite Nanocrystal Solids: Role of the Cold Carriers, Nanoconfinement, and the Surface. Nano Lett. 2020, 20, 2271–2278. 10.1021/acs.nanolett.9b04491. 32142303

[ref49] Carwithen B. P.; Hopper T. R.; Ge Z.; Mondal N.; Wang T.; Mazlumian R.; Zheng X.; Krieg F.; Montanarella F.; Nedelcu G.; Kroll M.; Siguan M. A.; Frost J. M.; Leo K.; Vaynzof Y.; Bodnarchuk M. I.; Kovalenko M. V.; Bakulin A. A. Confinement and Exciton Binding Energy Effects on Hot Carrier Cooling in Lead Halide Perovskite Nanomaterials. ACS Nano 2023, 17, 6638–6648. 10.1021/acsnano.2c12373. 36939330 PMC10100565

[ref50] Wang T.; Hopper T. R.; Mondal N.; Liu S.; Yao C.; Zheng X.; Torrisi F.; Bakulin A. A. Hot Carrier Cooling and Trapping in Atomically Thin WS_2_ Probed by Three-Pulse Femtosecond Spectroscopy. ACS Nano 2023, 17, 6330–6340. 10.1021/acsnano.2c10479. 36939760 PMC10100566

[ref51] Zhao J.; Zhang Q.; Sui L.; Niu G.; Zhang Y.; Wu G.; Yu S.; Yuan K.; Yang X. Evidence of Surface-Mediated Carrier-Phonon Scattering in MXene. ACS Nano 2023, 17, 23714–23722. 10.1021/acsnano.3c07431. 38009540

[ref52] Wang W.; Sui N.; Chi X.; Kang Z.; Zhou Q.; Li L.; Zhang H.; Gao J.; Wang Y. Investigation of Hot Carrier Cooling Dynamics in Monolayer MoS_2_. J. Phys. Chem. Lett. 2021, 12, 861–868. 10.1021/acs.jpclett.0c03110. 33428415

[ref53] Strait J. H.; Wang H.; Shivaraman S.; Shields V.; Spencer M.; Rana F. Very Slow Cooling Dynamics of Photoexcited Carriers in Graphene Observed by Optical-Pump Terahertz-Probe Spectroscopy. Nano Lett. 2011, 11, 4902–4906. 10.1021/nl202800h. 21973122

[ref54] Wang H.; Strait J. H.; George P. A.; Shivaraman S.; Shields V. B.; Chandrashekhar M.; Hwang J.; Rana F.; Spencer M. G.; Ruiz-Vargas C. S.; Park J. Ultrafast Relaxation Dynamics of Hot Optical Phonons in Graphene. Appl. Phys. Lett. 2010, 96, 081917 10.1063/1.3291615.

[ref55] Wang Y.; Wang Y.; Chen K.; Qi K.; Xue T.; Zhang H.; He J.; Xiao S. Niobium Carbide MXenes with Broad-Band Nonlinear Optical Response and Ultrafast Carrier Dynamics. ACS Nano 2020, 14, 10492–10502. 10.1021/acsnano.0c04390. 32687315

[ref56] Li G.; Natu V.; Shi T.; Barsoum M. W.; Titova L. V. Two-Dimensional MXenes Mo_2_Ti_2_C_3_T_z_ and Mo_2_TiC_2_T_z_: Microscopic Conductivity and Dynamics of Photoexcited Carriers. ACS Appl. Energy Mater. 2020, 3, 1530–1539. 10.1021/acsaem.9b01966.

[ref57] Shao Y.; He Q.; Xiang L.; Xu Z.; Cai X.; Chen C. Strengthened Optical Nonlinearity of V_2_C Hybrids Inlaid with Silver Nanoparticles. Nanomaterials 2022, 12, 1647 10.3390/nano12101647. 35630869 PMC9145371

[ref58] Mehata M. S.; Iimori T.; Yoshizawa T.; Ohta N. Electroabsorption Spectroscopy of 6-Hydroxyquinoline Doped in Polymer Films: Stark Shifts and Orientational Effects. J. Phys. Chem. A 2006, 110, 10985–10991. 10.1021/jp063927f. 16986830

[ref59] JALVISTE E.; OHTA N. Theoretical Foundation of Electroabsorption Spectroscopy: Self-Contained Derivation of the Basic Equations with the Direction Cosine Method and the Euler Angle Method. Journal of Photochemistry and Photobiology C: Photochemistry Reviews 2007, 8, 30–46. 10.1016/j.jphotochemrev.2007.01.001.

[ref60] El-Demellawi J. K.; Lopatin S.; Yin J.; Mohammed O. F.; Alshareef H. N. Tunable Multipolar Surface Plasmons in 2D Ti_3_C_2_T_x_ MXene Flakes. ACS Nano 2018, 12, 8485–8493. 10.1021/acsnano.8b04029. 30020767

[ref61] Tahini H. A.; Tan X.; Smith S. C. The Origin of Low Workfunctions in OH Terminated MXenes. Nanoscale 2017, 9, 7016–7020. 10.1039/C7NR01601H. 28534916

[ref62] Parker W. J.; Jenkins R. J.; Butler C. P.; Abbott G. L. Flash Method of Determining Thermal Diffusivity, Heat Capacity, and Thermal Conductivity. J. Appl. Phys. 1961, 32, 1679–1684. 10.1063/1.1728417.

[ref63] Zhang Q.; Chen Y.; Zhang Y.; Sun J.; Hu M.; Yan X.; Yuan K.; Yang X.; Li J. Surface Oxidation Modulates the Interfacial and Lateral Thermal Migration of MXene (Ti_3_C_2_T_x_) Flakes. J. Phys. Chem. Lett. 2020, 11, 9521–9527. 10.1021/acs.jpclett.0c02886. 33112154

[ref64] Huang Y.; Zhou J.; Wang G.; Sun Z. Abnormally Strong Electron–Phonon Scattering Induced Unprecedented Reduction in Lattice Thermal Conductivity of Two-Dimensional Nb_2_C. J. Am. Chem. Soc. 2019, 141, 8503–8508. 10.1021/jacs.9b01742. 31056905

[ref65] Gholivand H.; Fuladi S.; Hemmat Z.; Salehi-Khojin A.; Khalili-Araghi F. Effect of Surface Termination on the Lattice Thermal Conductivity of Monolayer Ti_3_C_2_T_z_ MXenes. J. Appl. Phys. 2019, 126, 065101 10.1063/1.5094294.

