# Supplementary material for: Ultrafast Carrier and Lattice Cooling in Ti2CTx MXene Thin Films
Source: Nano Lett. 2024 Nov 22;24(51):16333–41. doi: 10.1021/acs.nanolett.4c04583 (PMC11673571; doi:10.1021/acs.nanolett.4c04583)
Supplement: Supplementary file 1 — nl4c04583_si_001.pdf [file nl4c04583_si_001.pdf]

Supporting Information for

Ultrafast Carrier and Lattice Cooling in Ti_2CT_x MXene Thin Films

Tong Wang,[1] *Chengning Yao,*[1] *Ruoyu Gao,*[1] *Martin Holicky,*[1] *Beier Hu,*[1]*Sihui Liu,*[1] *Shuwei Wu,*[1] *Hyunho Kim,*[1] *Haoqing Ning,*[1]*Felice Torrisi,*[1,2] *Artem A. Bakulin*[1*]

[1]Department of Chemistry and Centre for Processible Electronics, Imperial College London, London W12 0BZ, United Kingdom

[2]Dipartimento di Fisica e Astronomia, Universita' di Catania and CNR-IMM (Catania Universita'), Via S. Sofia 64, 95123, Catania, Italy

*Corresponding author: a.bakulin@imperial.ac.uk;

Synthesis of Ti_2CT_x and $Ti_3C_2T_x$ MXene flakes:

The Ti_2AlC MAX phase is purchased from Laizhou Kai Kai Ceramic Materials Co., Ltd, and Ti_3AlC_2 MAX phase is purchased from Carbon-Ukraine Ltd. HCl (12 M, 14.0 mL for $Ti_3C_2T_x$ and 11.2 mL for Ti_2CT_x) was added into deionized water (6.0 mL for $Ti_3C_2T_x$ and 4.8 mL for Ti_2CT_x) in a vented polyethylene (HDPE) container, followed by adding LiF (1.5 g for $Ti_3C_2T_x$ and 1.0 g for Ti_2CT_x) powder into the solution.[1] The mixture was stirred for 5 mins using a magnetic polytetrafluoroethylene (PTFE) bar to fully dissolve the salt. MAX (1.0 g for $Ti_3C_2T_x$ and 0.9 g for Ti_2CT_x) powder was then carefully added to the solution over the course of 10 mins to avoid initial overheating by aggressive exothermal reactions. The container was then immersed in a silicon oil bath (at 45 °C for 24 hours for $Ti_3C_2T_x$ and at 25 °C for 15 hours for Ti_2CT_x) to allow a fully selective Aluminium etching. After the etching process, the mixture was washed for 3-4 cycles via centrifugation (GT2R centrifuge, Fisher, TX-400 rotor) until the pH of the supernatant reached approximately 6. In each washing cycle, the solution was added with deionized water and centrifuged at 800 ~ 3000 rcf for 5 mins, after which the supernatant was discarded, and the sediment was redispersed in deionized water. The fully washed mixture was manually shaken (minimally intensive layer delamination, MILD method) to allow delamination of MXene flakes and centrifuged at 800 rcf for 10 mins. The obtained supernatant was collected as the MXene suspension for further characterization and thin-film deposition.[2]

Preparation of MXene films:

The MXene films were deposited by spray coating. The MXene suspension was diluted to 1.0 mg mL^{-1} and was sprayed onto a pre-cleaned glass using an airbrush (Everything, AB-130, 0.25-mm nozzle, 1.5 bar N_2 flow). The nozzle-glass distance was kept at 15 cm and the thickness of the film was dependent on the amount of suspension used for spray.

Raman spectra of MXenes compared to MAX:

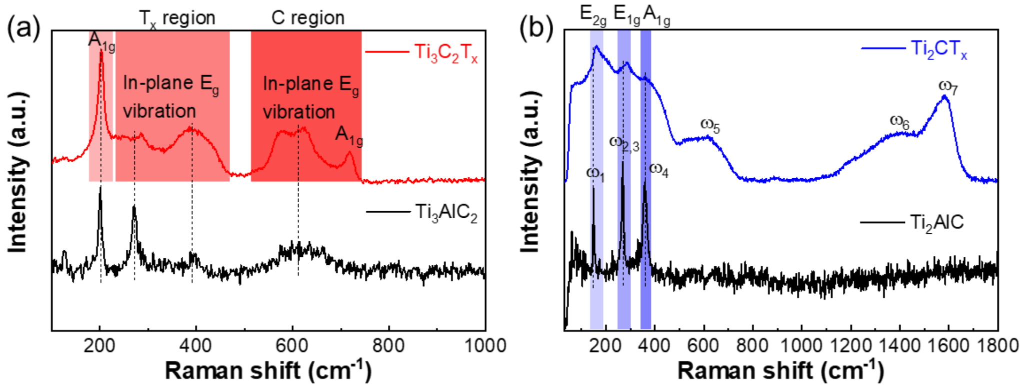

Figure S1. *(a) Raman spectra of $Ti_3C_2T_x$ (red curve) and Ti_3AlC_2 (black curve), (b) Raman spectra of Ti_2CT_x (blue curve) and Ti_2AlC (black curve).*

Figure S1(a) shows the Raman spectra of $Ti_3C_2T_x$ (red curve) and Ti_3AlC_2 (black curve). Raman spectra of the $Ti_3C_2T_x$ MXene shows two broad resonant regions, denoted as the T_x (functional groups) region (between ~223 and ~480 cm^{-1}) and the C region (between ~503 and ~750 cm^{-1}).[3] The convoluted peaks in the T_x region originate from the in-plane E_g vibrations of the T_x, while the convoluted peaks in the C region are caused by both in-plane E_g vibrations and out-of-plane A_{1g} vibrations of the carbon atoms. An additional peak appears at ~205 cm^{-1} caused by an out-of-plane A_{1g} vibrational mode that originates from the carbon atoms, two Ti layers and functional surface groups of ─OH, ─O, and ─F.[4]

Figure S1(b) shows the Raman spectra of Ti_2CT_x (blue curve) and Ti_2AlC (black curve). The peaks observed for Ti_2AlC (ω_1, $\omega_{2,3}$ and ω_4) correlates to the Raman active modes of E_{2g}, E_{1g}, and A_{1g}, respectively,[5] which originate from the shear and longitudinal vibrations of the Ti and the Al atoms.[6] The slightly weak broad peak (ω_5) at ~616 cm^{-1} may be attributed to non-stochiometric TiC_x.[6] The peaks of Ti_2CT_x are upshifted and broadened, which indicates the successful etching of the Al atom from Ti_2AlC phase (as validated from XRD patterns) and the T_x termination.[7] Two peaks (ω_6 and ω_7) observed at 1340 cm^{-1} and 1576 cm^{-1} originate

from the D and G bands of amorphous carbon, respectively, which indicates the existence of carbon in the disordered Ti_2CT_x after the etching and exfoliation process.[8]

XRD patterns of MXenes compared to MAX:

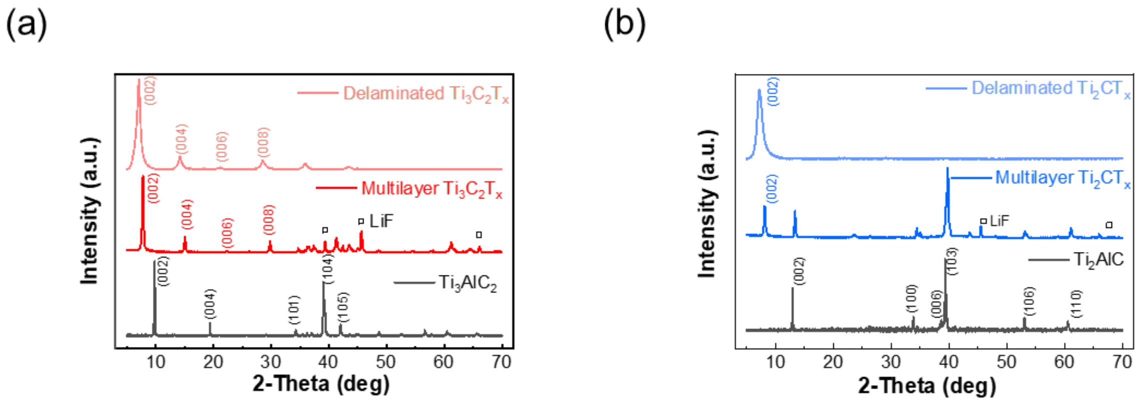

Figure S2. *(a) XRD patterns of delaminated $Ti_3C_2T_x$ (light red, after MILD exfoliation), multilayer $Ti_3C_2T_x$ (dark red, after selective etching) and Ti_3AlC_2 (black), (b) XRD patterns of delaminated Ti_2CT_x (light blue, after MILD exfoliation), multilayer Ti_2CT_x (dark blue, after selective etching) and Ti_2AlC (black).*

Figure S2(a) presents the XRD patterns of delaminated $Ti_3C_2T_x$ (light red, after MILD exfoliation), multilayer $Ti_3C_2T_x$ (dark red, after selective etching) and Ti_3AlC_2 (black). The downshift of the first (002) peak of the multilayer $Ti_3C_2T_x$ at $2\theta = 7.7$ ° compared to that of Ti_3AlC_2 at $2\theta = 10$ ° indicates the successful etching of the Al atom.[1] The further downshift of the first (002) peak ($2\theta = 7.2$ °) of delaminated $Ti_3C_2T_x$ compared to that of multilayer $Ti_3C_2T_x$ revealed the restacked structure of MXene nanosheets have more ─OH groups to form a higher hydration state. The (002) characteristic peak of delaminated $Ti_3C_2T_x$ is also widened, indicating that the interlayer spacing became larger after the MILD exfoliation.

Figure S2(b) presents the XRD patterns of delaminated Ti_2CT_x (light blue, after MILD

exfoliation), multilayer Ti_2CT_x (dark blue, after selective etching) and Ti_2AlC (black). The downshift of the first (002) peak of the multilayer Ti_2CT_x ($2\theta = 8.1$ º), and the further downshift of the delaminated Ti_2CT_x ($2\theta = 7.2$ º) compared to that of Ti_2AlC ($2\theta = 13$ º) indicates the successful etching of the Al atom and delamination of the MXene.[7] The residue of Ti_2AlC phase in the multilayer Ti_2CT_x indicates the partially etched MAX phase.

Atomic Force microscopy of MXene flakes:

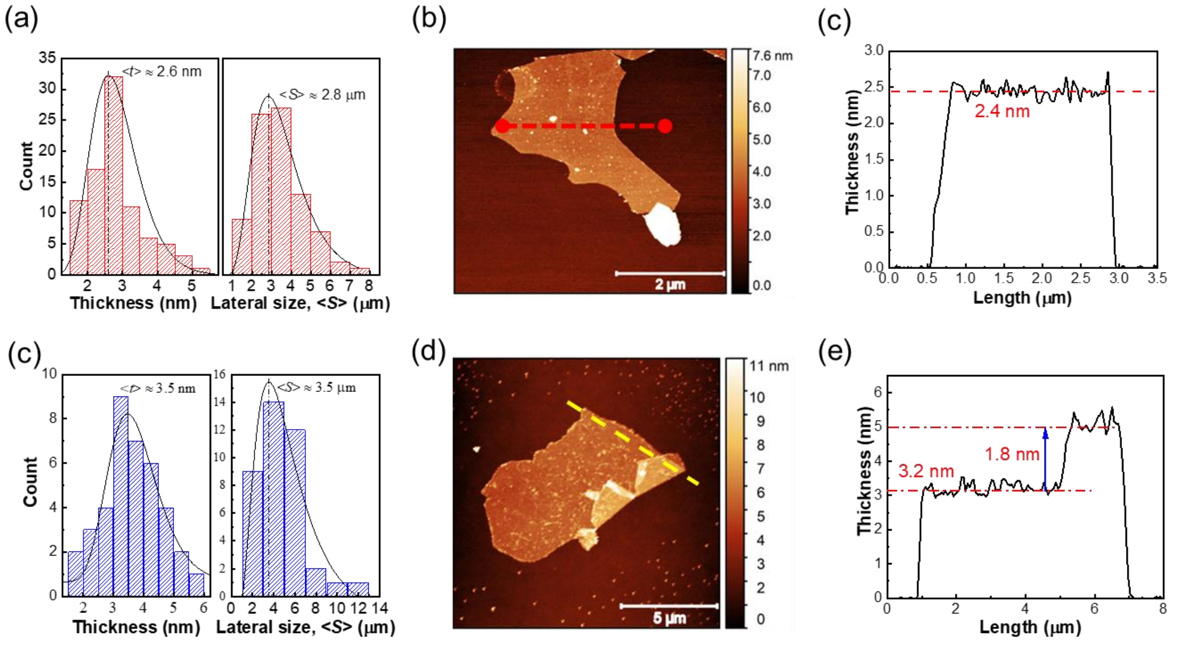

Figure S3. *(a) AFM statistics on the thickness and lateral size over 89 $Ti_3C_2T_x$ flakes, (b) a typical monolayer $Ti_3C_2T_x$ flake and (c) its height profile over the lateral length; (d) AFM statistics on the thickness and lateral size over 38 Ti_2CT_x flakes, (e) a typical monolayer Ti_2CT_x flake and (f) its height profile over the lateral length.*

AFM statistics in Figure S3 is used to estimate the thickness ($<t>$) and lateral size ($<S>$) distribution of the delaminated Ti_2CT_x MXene flakes ($<t_{Ti2CTx}>$ and $<S_{Ti2CTx}>$) and $Ti_3C_2T_x$ MXene flakes ($<t_{Ti3C2Tx}>$ and $<S_{Ti3C2Tx}>$). AFM statistics were collected separately from 89

$Ti_3C_2T_x$ flakes and 38 Ti_2CT_x flakes, respectively. The $<S>$ is obtained via $<S> = xy^{0.5}$, where x and y refer to the length and width of a particular flake. Log-normal distributions are applied to fit the AFM statistics, with the peak values as the average $<t>$ and $<S>$. AFM was performed using the Keysight 5500 SPM AFM (Keysight Technologies). Measurements were collected over a 20 μm × 20 μm area at a scanning rate of 0.40 Hz using a point probe (Silicon-SPM-Sensor, PPP-NCLR-50, Nano-sensors) operating in a tapping mode. The ink was diluted at a factor of 1000 and then drop-casted onto a pre-cleaned Si/SiO_2 substrate.

Figure S3a shows that the distribution of $<t_{Ti3C2Tx}>$ and $<S_{Ti3C2Tx}>$ both follow a log-normal distribution peaked at 2.6 nm and 2.8 μm, respectively. The results indicate the as-prepared $Ti_3C_2T_x$ solutions consists of ~ 70% monolayer and bilayer flakes, considering a monolayer thickness of 1.6 nm and additional thickness attributed to the 1-nm water molecules trapped between the flake and the substrate.[2,9] Figure S3b and S3c present a high-resolution AFM image for a monolayer $Ti_3C_2T_x$ flake with a $< t_{Ti3C2Tx} >$ of 2.4 nm and a $< S_{Ti3C2Tx} >$ of 2.5 μm.

Figure S3d shows that the distribution of $<t_{Ti2CTx}>$ and $<S_{Ti2CTx}>$ also follow a log-normal distribution peaked at 3.5 nm and 3.5 μm, respectively. Figure S3e and S3f present a high-resolution AFM image for a folded Ti_2CT_x flake with a $< t_{Ti2CTx} >$ of 3.2 nm and a $<S_{Ti2CTx} >$ of 6.6 μm. The thickness of the folded edge of the flake is 1.8 nm, which indicates it is a monolayer. The additional thickness of 1.4 nm can be attributed to the water molecular trapped between the flake and the substrate.

Details of fs-transient absorption spectroscopy setup:

The TA experiments are based on a commercial Helios transient absorption spectroscopy setup (Spectra Physics, Newport Corp.). Ultrafast 800 nm (1 kHz, <100 fs pulse duration) laser pulses were generated by a Ti:sapphire regenerative amplifier (Solstice, Spectra Physics, Newport Corp.). This fundamental output is split into two portions. The first portion is fed into an optical parametric amplifier (TOPAS Prime, Light Conversion) and a frequency mixer (Niruvis, Light Conversion) for the pump pulse. The remaining 800 nm pulses are fed into a sapphire crystal to generate the broadband visible light probe. The two beams are focused onto the sample at the same spot (diameter ~0.5 mm), and the transmitted probe is collected by a CCD spectrometer. To mitigate shot-to-shot noise, we use another CCD spectrometer to measure fluctuations in a reference beam that is split off from the probe before it hits the sample by a neutral density filter. A mechanical chopper in the pump beam is modulated at 500 Hz to block every other pulse and relay the TA signal.

The sample was housed in a N_2-purged quartz cuvette during measurements at room temperature. In temperature dependent measurement, the samples were measured within the crystat (OptistatDN-V, Oxford Instruments) under high vacuum. The temperature was controlled by the temperature controller (MercuryiTC).

Details of nanosecond pump-probe (ns-PP) spectroscopy setup:

The pump pulse (1064 nm, ~15 ns) was generated from an Nd:YAG laser (Innolas), which is synchronised to a Yb:KGW laser (Pharos, 1030 nm, Light Conversion) at 5 kHz. A mechanical chopper in the pump beam is modulated at 2.5 kHz to block every other pulse and relay the pump-probe signal. The diameter of the spot size of the pump beam is around 0.3 mm. The probe beam was generated through a nonlinear optical parametric amplifier (NOPA, ORPHEUS-N, Light Conversion) pumped by the Yb:KGW laser at 500 nm and 630 nm. The time delay between the pump and probe pulse was achieved by an electrical delay generator (DG645, Stanford Research Systems). Changes to the transmission of the probe were detected by an amplified Si photodetector (PDA36A2, ThorLabs), coupled to a lock-in amplifier (MFLI, Zurich Instruments). A short pass filter (FESH0800, ThorLabs) was mounted on the amplified Si photodetector to filter out the scattered pump beam. The sample was housed in a N_2-purged quartz cuvette during measurements.

Kinetics of $Ti_3C_2T_x$ MXene with different fluence, excitation wavelength, and environment temperature:

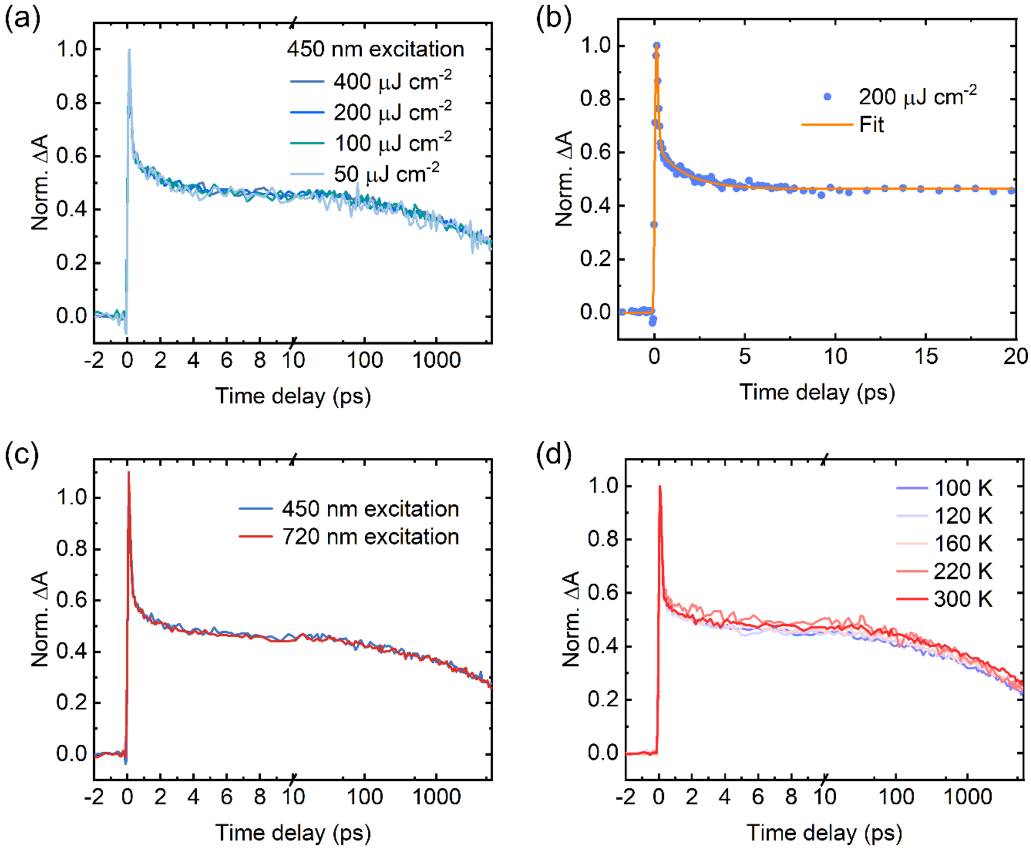

Figure S4 Ultrafast kinetics of $Ti_3C_2T_x$ MXene film probed around 500 nm. *(a) Kinetics of $Ti_3C_2T_x$ MXene film excited at 450 nm at different fluences. (b) Fit of the kinetics excited at 450 nm (200 μJ cm^{-2}) by Eq. 1. (c) Comparison of the kinetics between excitation at 450 nm (200 μJ cm^{-2}, in blue) and 720 nm (350 μJ cm^{-2}, in red). (d) Normalized kinetics of $Ti_3C_2T_x$ MXene film excited at 450 nm at different environment temperatures.*

The carrier cooling time of $Ti_3C_2T_x$ is 1.6 ± 0.2 ps, obtained by fitting by Eq. 1 in the main text. The dynamics of $Ti_3C_2T_x$ does not show significant change with pump fluence, pump wavelength and environmental temperatures. No evidence of hot-phonon bottleneck is observed in $Ti_3C_2T_x$. Similar results have been observed by Zhao et al.[10] However, the e-e scattering in $Ti_3C_2T_x$ is independent on the excited carrier density, different from Ti_2CT_x. This difference can be caused by different electron mobilities in two MXenes.

Normalised TA spectra of Ti_2CT_x and $Ti_3C_2T_x$ MXene films:

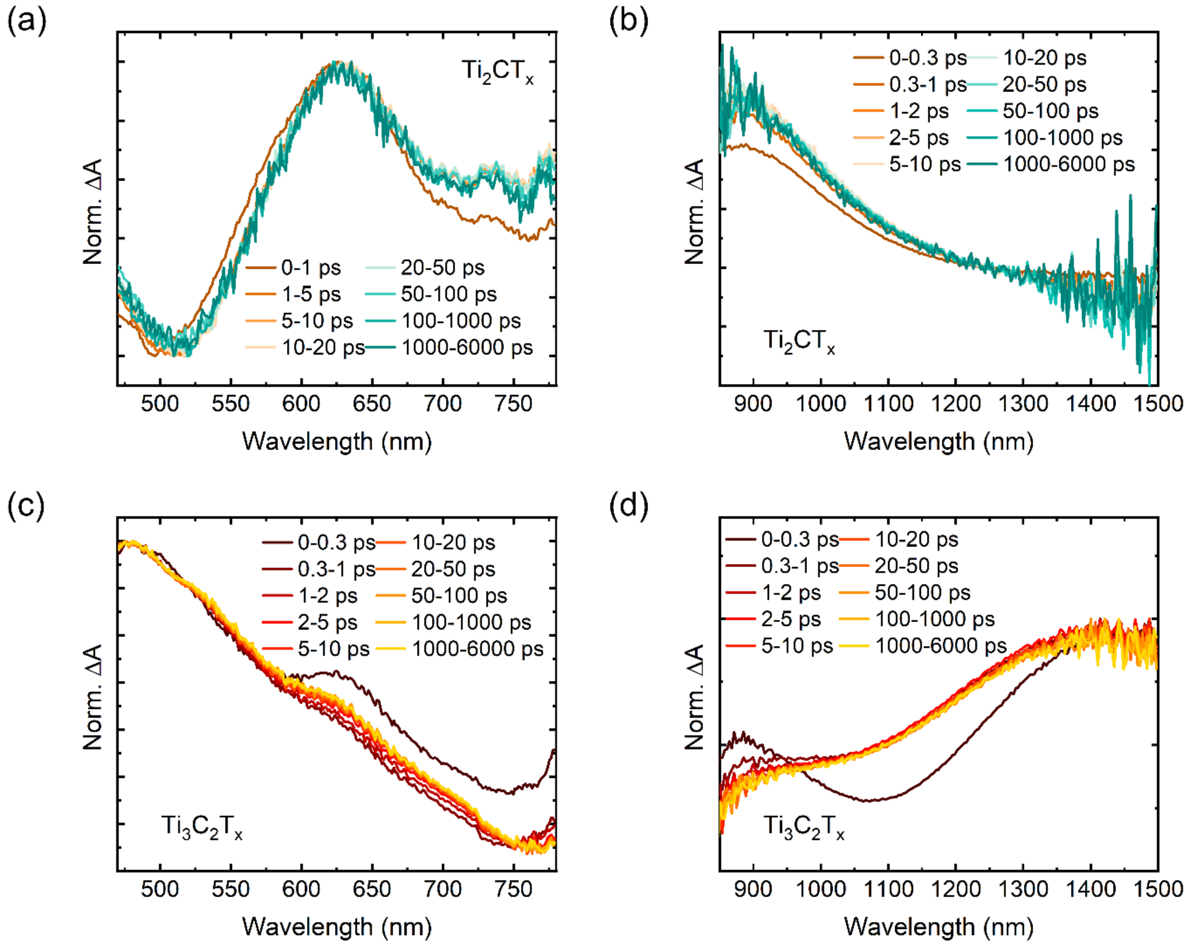

Figure S5. *Normalised TA spectra of Ti_2CT_x and $Ti_3C_2T_x$ MXene films with 450 nm excitation in the visible and NIR region.*

The normalised TA spectra of Ti_2CT_x and $Ti_3C_2T_x$ MXene films (Figure S5) show constant spectral shape in both visible and NIR region after 1 ps. The different spectral shape within 1 ps can be caused by e-e scattering, Stark effect, and other coherent artefacts.

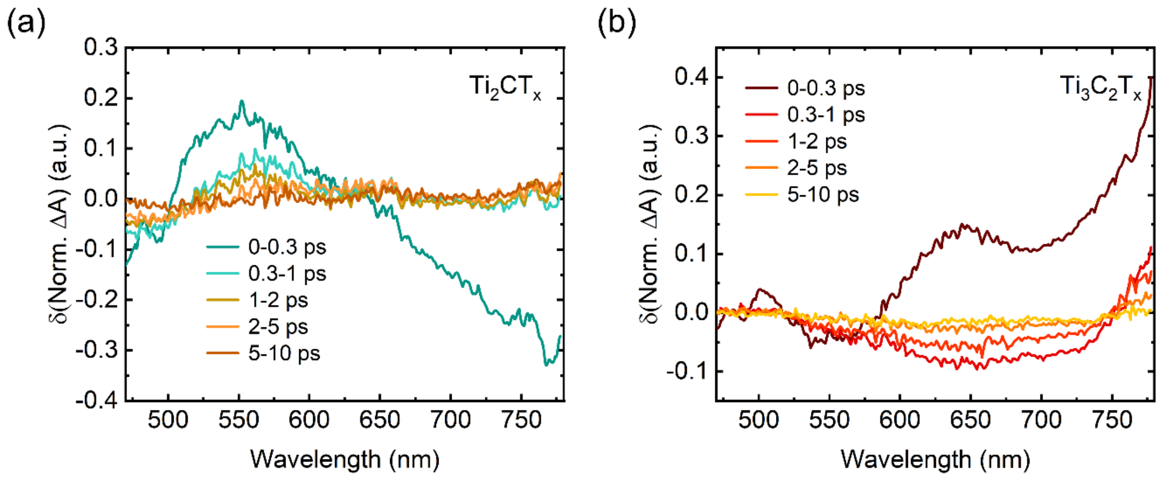

Figure S6. TA signal difference at early times.

As shown in Figure S5, the normalized TA spectra at long delay times (>10 ps) are identical, which is attributed to a hot lattice after reaching thermal equilibrium with hot carriers. However, the differences between the spectra at early times (<10 ps) and those at late times (>100 ps) are not clearly visible in Figure S5. To better illustrate the subtle spectral evolutions at early delay times, we normalized the TA spectra and subtracted the normalized TA spectra at late times (100 ps to 1 ns) from those at early times, denoted as δ(ΔA), as shown in Figure S6. In both MXenes, the TA spectral differences evolve within 5 ps (i.e., δ(ΔA) is non-zero), and no differences are observed after 10 ps (i.e., δ(ΔA) approaches zero). Furthermore, for both MXenes, the δ(ΔA) spectra within the first 300 fs differ from those observed between 0.3 ps and 5 ps. The resulting δ(ΔA) spectra within the first 300 fs are attributed to electron-electron scattering and hot carriers, while the δ(ΔA) spectra from 0.3 to 5 ps represent signals induced by hot carriers towards reaching thermal equilibrium with the lattice.

Steady-state absorption spectra of Ti_2CT_x and $Ti_3C_2T_x$ MXene films at different temperatures:

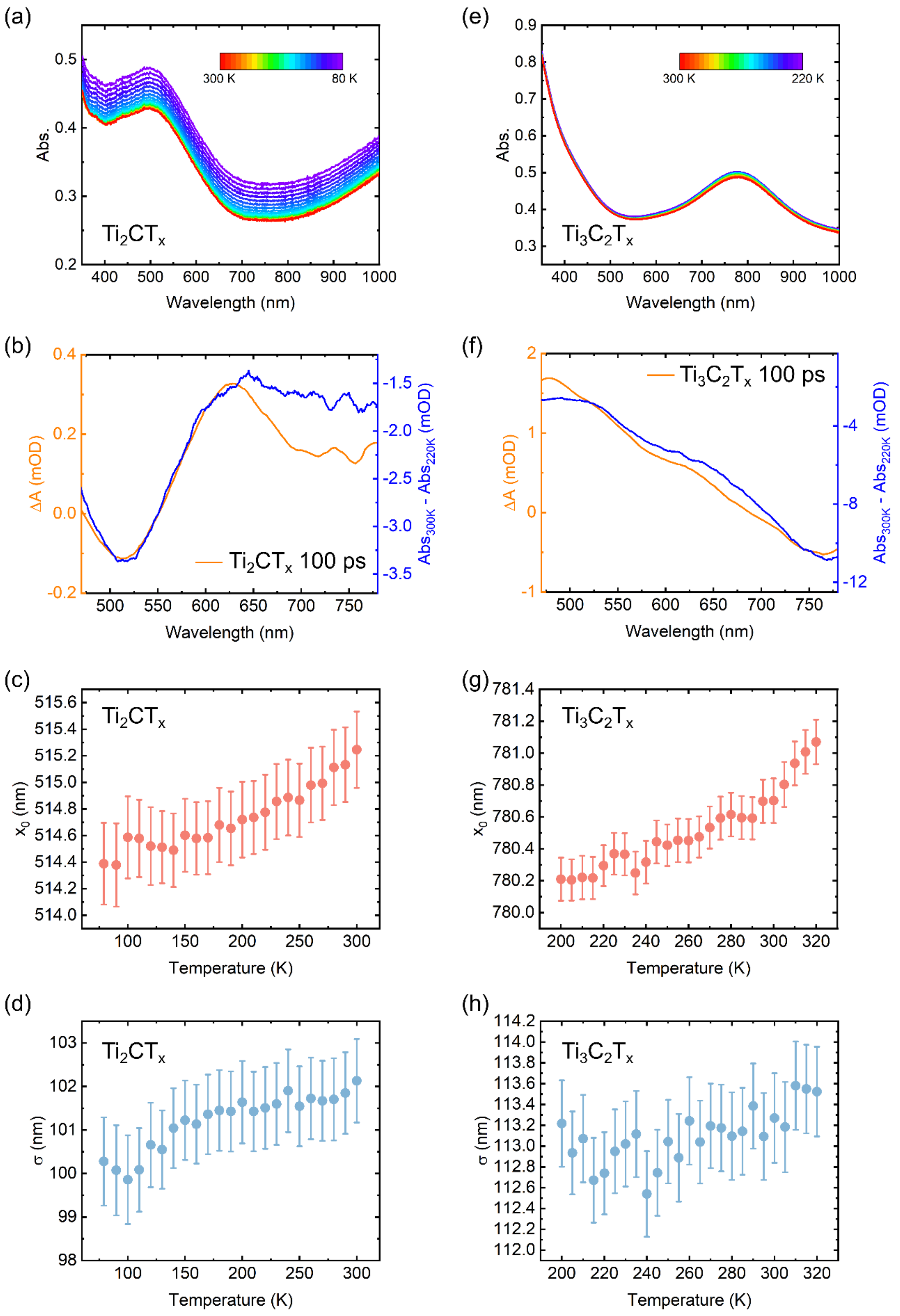

Figure 7. *(a) and (e) Steady-state absorption spectra for* Ti_2CT_x *and* $Ti_3C_2T_x$ *MXene films at different temperatures. (b) and (f) comparison between TA spectra at 100 ps and difference of absorption spectra at two 300 K and 220 K of* Ti_2CT_x *and* $Ti_3C_2T_x$ *MXene films. (c-d) and (g-h) The position and width of plasmonic resonant peak at different temperatures for* Ti_2CT_x *and* $Ti_3C_2T_x$ *MXene films.*

To prevent the MXene films from degradation, we measured the steady-state absorption spectra for Ti_2CT_x and $Ti_3C_2T_x$ MXene films at lower temperatures.

Figure S7(a) and (e) plots the steady-state absorption spectra for Ti_2CT_x and $Ti_3C_2T_x$ MXene films at different temperatures. We calculate the difference of absorption spectra at two 300 K and 220 K (i.e., Abs_{300K}-Abs_{220K}) as an analogue of the TA results. Figure S7 (b) and (f) shows that the TA spectral shape at 100 ps can be almost described as the difference of absorption spectra at two 300 K and 220 K of Ti_2CT_x and $Ti_3C_2T_x$ MXene films. This similarity enables us to speculate the TA spectra of both MXene films origin from thermochromic effect. However, for both MXenes, baselines are needed to fully describe the TA spectra with the difference of absorption spectra at two different temperatures. The TA spectra show positive (PIA) and negative (GSB) signals in the spectral window, but the absorption spectra at two different temperatures only shows negative results. Although similar spectral shape comparison between TA signal and difference of steady-state absorption spectra has been done by Zhang et al. and Li et al., the sign of the difference of steady-state absorption spectra is not provided in their results.[11,12] Therefore, we choose to use the linear combination of first and second derivatives of the steady-state absorption spectra of MXenes to represent the respective TA spectra. This method allows us to explain the origins of the TA spectral shape.

The plasmonic resonant peaks are fitted by a Lorentzian function with a linear baseline, shown in Eq. S1.

$$y = H\frac{\sigma}{(x - x_0)^2 + \sigma^2} + ax + b \qquad \text{(Eq. S1)}$$

where H is the peak height, σ is the peak width, x_0 is the peak center position, and a and b are parameters for the linear baseline.

The extracted x_0 and σ for both MXenes films as a function of temperature is shown in Figure S7 (c-d) and (g-h). As shown in Figure S7 (c) and (d), the plasmonic resonant peak for Ti_2CT_x MXene film is red-shifted and broadened. In Figure S7 (g) and (h), the plasmonic resonant peak for $Ti_3C_2T_x$ MXene film is red-shifted, but the peak width at different temperatures keeps almost constant. These spectral changes in both MXenes are similar to the analysis of TA spectra with the derivatives of the steady-state absorption spectra.

Differentials of the steady-state absorption spectrum of Ti_2CT_x and $Ti_3C_2T_x$ MXene films:

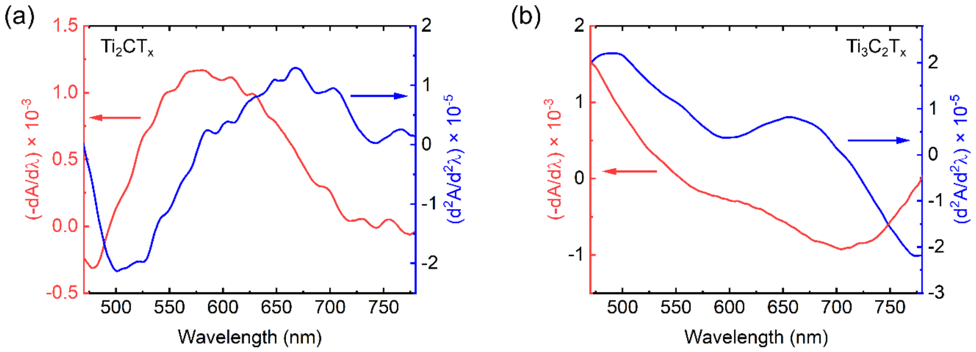

Figure S8. *The first (in red) and second (in blue) derivatives of the steady-state absorption spectrum of Ti_2CT_x (shown in a) and $Ti_3C_2T_x$ (shown in b) MXene film.*

ns-PP kinetics of MXene films with different fluence:

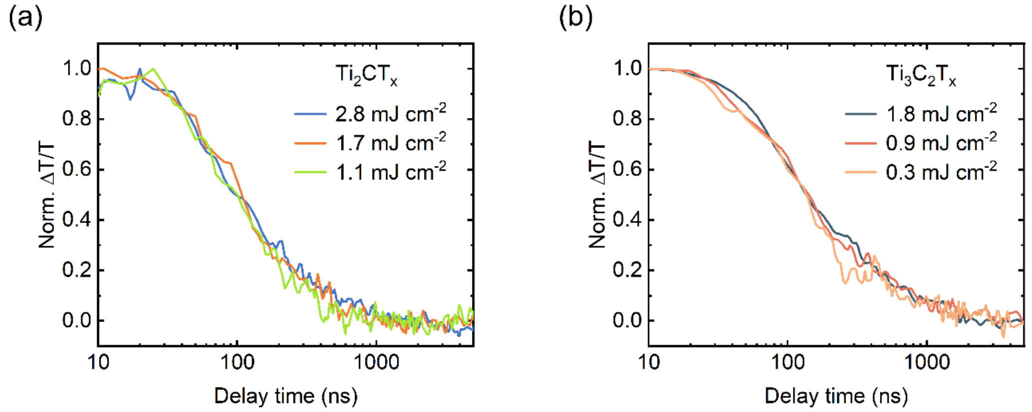

Figure S9. *ns-PP kinetics of Ti_2CT_x and $Ti_3C_2T_x$ MXene films at different fluences under 1064 nm excitation.*

Thermal diffusion in MXene film:

We use a one-dimensional (1D) thermal diffusion model to fit the ns-PP kinetics of MXene films to extract the thermal diffusivity. Here we assume a continuous medium for simplicity, which is an acceptable assumption given that individual chemical vapor deposition (CVD)-grown $Ti_3C_2T_x$ flakes and networks of individual $Ti_3C_2T_x$ flakes have shown to have the same electronic transport.[3] We model the relative temperature difference caused by the incident laser pulse between the MXene film and the ambient environment, rather than the absolute temperature of the MXene film. In the kinetics analysis, the time for thermal equilibrium between hot electrons and lattice is a few picoseconds and independent on fluence. Therefore, we assign the initial temperature distribution as the same as the distribution of absorbed photon number. We use Dirichlet condition as the other boundary condition. All equations are given as follow:

$$\frac{\partial T(x,t)}{\partial t} = \alpha \frac{\partial^2 T(x,t)}{\partial x^2} \qquad \text{(Eq. S2)}$$

$$T(x,0) = ne^{-\frac{x}{a}} \qquad \text{(Eq. S3)}$$

$$\frac{\partial T(x_0,t)}{\partial t} = 0 \qquad \text{(Eq. S4)}$$

$$T(x_{max},t) = 0 \qquad \text{(Eq. S5)}$$

where T is the temperature difference from room temperature, t is the delay time, x is the thickness of the sample, α is thermal diffusivity, n is the initial temperature, which is linearly proportional to the incident photon number, a is the absorption coefficient, x_0 is the interface between the MXene thin film and N_2 atmosphere, and x_{max} is the interface between the MXene thin film and the glass substrate. Eq. S5 is valid under assumption that thermal diffusivity of MXene is much lower than that of glass substrate; further calculations confirm this assumption is correct. The equations are solved by pdepe solver function in MATLAB. We take the integral

$T(t)$ as a function of x, $\int T(t)\ dx$, as the fitting function of the ns-PP kinetics.

The absorption coefficient, a, is determined from the absorption at 1064 nm for both MXenes films by Eq. S5, shown in Table S1.

$$\alpha = \frac{\text{Absorbance}}{thickness} * 2.303 \qquad \text{(Eq. S5)}$$

Table S1. *Absorbance and absorption coefficient of MXene films*

**Ti_2CT_x MXene 120 nm**		**$Ti_3C_2T_x$ MXene 110 nm**	
Absorbance	a (1/nm)	Absorbance	a (1/nm)
0.406	0.008	0.548	0.011

Figure S10(a) shows the simulated $\int T(t)\ dx$ at fixed α (0.06 mm^2/s) is independent on the initial temperature, n, which further supports the pump power independency of the ns-PP kinetics. Therefore, for simplicity, the n is fixed as 1000 K for the fitting of the ns-PP data. The simulated 3D colourmap of T as a function of x and t with a thickness of 120 nm is shown in Figure S10(b). It is noted that the thermal transfer to the N_2 atmosphere from the MXene film is not considered in the 1D model. In addition, the in-plane heat dissipation is not considered in the 1D thermal diffusion model. Therefore, there might be heat loss during the test, which means the estimated thermal diffusivity values for both MXenes films might be overestimated.

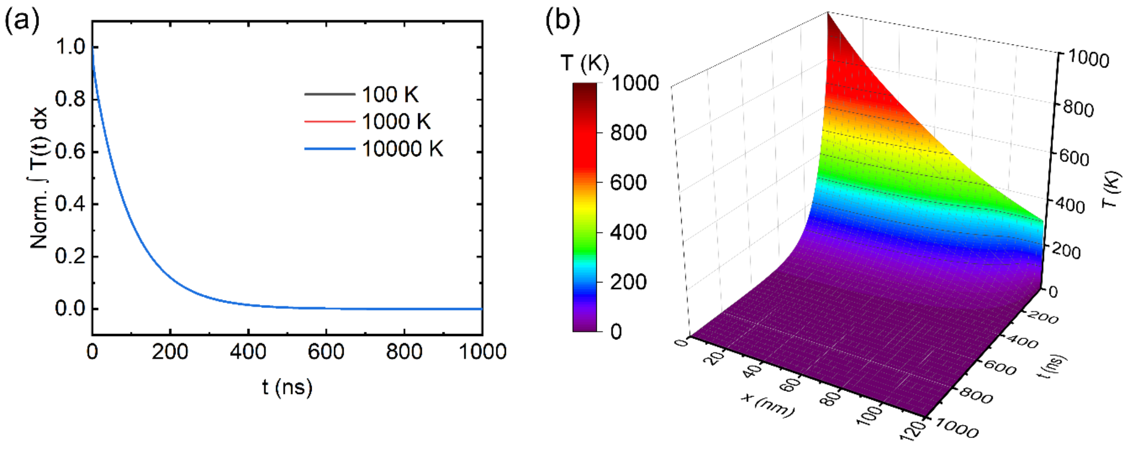

Figure S10. *(a) Simulated* $\int T(t)\,dx$ *with different initial temperatures. (b) 3D colourmap of* T *with a thickness of 120 nm.*

References:

(1) Yong, S.; Yao, C.; Hillier, N.; Kim, H.; Holicky, M.; Liu, S.; Doherty, R.; Torrisi, F.; Beeby, S. Ti_3C_2 MXene as Additive for Low-Cost Textile Supercapacitors with Enhanced Electrical Performance. *Adv Mater Technol* **2024**, *9*, 2301266.

(2) Lipatov, A.; Alhabeb, M.; Lukatskaya, M. R.; Boson, A.; Gogotsi, Y.; Sinitskii, A. Effect of Synthesis on Quality, Electronic Properties and Environmental Stability of Individual Monolayer Ti_3C_2 MXene Flakes. *Adv Electron Mater* **2016**, *2*, 1600255.

(3) Piatti, E.; Arbab, A.; Galanti, F.; Carey, T.; Anzi, L.; Spurling, D.; Roy, A.; Zhussupbekova, A.; Patel, K. A.; Kim, J. M.; Daghero, D.; Sordan, R.; Nicolosi, V.; Gonnelli, R. S.; Torrisi, F. Charge Transport Mechanisms in Inkjet-Printed Thin-Film Transistors Based on Two-Dimensional Materials. *Nat Electron* **2021**, *4*, 893–905.

(4) Kang, R.; Zhang, Z.; Guo, L.; Cui, J.; Chen, Y.; Hou, X.; Wang, B.; Lin, C.-T.; Jiang, N.; Yu, J. Enhanced Thermal Conductivity of Epoxy Composites Filled with 2D Transition Metal Carbides (MXenes) with Ultralow Loading. *Sci Rep* **2019**, *9*, 9135.

(5) Presser, V.; Naguib, M.; Chaput, L.; Togo, A.; Hug, G.; Barsoum, M. W. First-order Raman Scattering of the MAX Phases: Ti_2AlN, $Ti_2AlC_{0.5}N_{0.5}$, Ti_2AlC, $(Ti_{0.5}V_{0.5})_2AlC$, V_2AlC, Ti_3AlC_2, and Ti_3GeC_2. *Journal of Raman Spectroscopy* **2012**, *43*, 168–172.

(6) Melchior, S. A.; Raju, K.; Ike, I. S.; Erasmus, R. M.; Kabongo, G.; Sigalas, I.; Iyuke, S. E.; Ozoemena, K. I. High-Voltage Symmetric Supercapacitor Based on 2D Titanium Carbide (MXene, Ti_2CT_x)/Carbon Nanosphere Composites in a Neutral Aqueous Electrolyte. *J Electrochem Soc* **2018**, *165*, A501–A511.

(7) Liu, F.; Zhou, A.; Chen, J.; Zhang, H.; Cao, J.; Wang, L.; Hu, Q. Preparation and

Methane Adsorption of Two-Dimensional Carbide Ti2C. *Adsorption* **2016**, *22*, 915–922.

(8) Lai, S.; Jeon, J.; Jang, S. K.; Xu, J.; Choi, Y. J.; Park, J.-H.; Hwang, E.; Lee, S. Surface Group Modification and Carrier Transport Properties of Layered Transition Metal Carbides (Ti_2CT_x, T: –OH, –F and –O). *Nanoscale* **2015**, *7*, 19390–19396.

(9) Carey, T.; Cacovich, S.; Divitini, G.; Ren, J.; Mansouri, A.; Kim, J. M.; Wang, C.; Ducati, C.; Sordan, R.; Torrisi, F. Fully Inkjet-Printed Two-Dimensional Material Field-Effect Heterojunctions for Wearable and Textile Electronics. *Nat Commun* **2017**, *8*, 1202.

(10) Zhao, J.; Zhang, Q.; Sui, L.; Niu, G.; Zhang, Y.; Wu, G.; Yu, S.; Yuan, K.; Yang, X. Evidence of Surface-Mediated Carrier-Phonon Scattering in MXene. *ACS Nano* **2023**, *17,* 23714–23722.

(11) Zhang, Q.; Yan, L.; Yang, M.; Wu, G.; Hu, M.; Li, J.; Yuan, K.; Yang, X. Ultrafast Transient Spectra and Dynamics of MXene ($Ti_3C_2T_x$) in Response to Light Excitations of Various Wavelengths. *The Journal of Physical Chemistry C* **2020**, *124,* 6441–6447.

(12) Li, J.; Zhang, Q.; Yan, L.; Wu, G.; Hu, M.; Lin, X.; Yuan, K.; Yang, X. Ultrafast Flash Energy Conductance at MXene-Surfactant Interface and Its Molecular Origins. *Adv Mater Interfaces* **2019**, *6*, 1–8.
